# Stability of Begomoviral pathogenicity determinant βC1 is modulated by mutually antagonistic SUMOylation and SIM interactions

**DOI:** 10.1186/s12915-020-00843-y

**Published:** 2020-08-31

**Authors:** Ashwin Nair, Kiran Sankar Chatterjee, Vikram Jha, Ranabir Das, P. V. Shivaprasad

**Affiliations:** 1 National Centre for Biological Sciences, Tata Institute of Fundamental Research, GKVK Campus, Bellary Road, Bangalore, 560065 India; 2 SASTRA University, Thirumalaisamudram, Thanjavur, 613401 India; 3 Present address: BIOSS Centre for Biological Signalling Studies, Faculty of Biology, Albert-Ludwigs-Universitaet Freiburg, 79104 Freiburg im Breisgau, Germany

**Keywords:** βC1 protein, SUMOylation, SIM interaction, Begomovirus, Host-pathogen interactions, Post-translational modification, NMR

## Abstract

**Background:**

To successfully invade new hosts, plant viruses must break host resistance and be competent to move within and between plant cells. As a means, viral proteins known as pathogenicity determinants have evolved to coordinate a network of protein interactions. The βC1 protein encoded by specific geminiviral satellites acts as a key pathogenicity determinant for this disease-causing family of plant viruses. Post-translational modifications (PTMs) such as ubiquitination and phosphorylation of the βC1 protein have been shown to occur in diverse viruses. However, the relevance of these and other layers of PTMs in host-geminiviral interactions has not been fully understood.

**Results:**

Here we identified the significance of a novel layer of PTMs in the βC1 protein of Synedrella yellow vein clearing virus (SyYVCV), a newly identified member of the Begomovirus genus of Geminiviruses. This protein has conserved SUMOylation and SUMO-interacting motifs (SIMs), and we observed SUMOylation of SyYVCV βC1 in host plants as a defensive strategy against ubiquitin-mediated degradation. Counteracting this, SIMs encoded in βC1 mediate the degradation of βC1; however, both these PTMs are essential for the function of βC1 protein since SIM and SUMOylation motif mutants failed to promote pathogenicity and viral replication in vivo. SUMOylation in different motifs of βC1 led to functionally distinct outcomes, regulating the stability and function of the βC1 protein, as well as increased global SUMOylation of host proteins.

**Conclusion:**

Our results indicate the presence of a novel mechanism mediating a fine balance between defence and counter-defence in which a SIM site is competitively sought for degradation and, as a counter-defence, βC1 undergoes SUMOylation to escape from its degradation.

## Background

Viruses are obligate intracellular pathogens overcoming host defence as a means of survival. During millions of years of evolution, many strategies have been evolved by both the host and the virus to counter each other [1]. The dependence of viruses on host resources, along with the variations in the host defence, results in acute susceptibility, chronic infections or resistance. Within the constraints of a small genetic material, viruses code for essential proteins that are mostly multifunctional, playing critical roles in viral replication, packaging and counter-defence. A few proteins termed as pathogenicity determinants have a special place since they are essential to counter host defences, thereby playing crucial roles in viral infection. Often, viruses may replicate without these proteins, but are unable to mount a systemic infection, eventually being subdued by the host defence system [2–6].

The host has evolved and modified its innate cellular pathways to detect and neutralize various pathogenic threats apart from maintaining regular cellular homeostasis [7]. Post-translational modification (PTM) of proteins diversifies their functions as well as offering an additional regulation over their cellular activity. The host has evolved its PTM machinery to modify and subdue incoming pathogenic proteins to affect pathogenicity [7]. For example, the host recognizes and directs phosphorylation of Turnip yellow mosaic virus (TYMV) RNA-dependent DNA polymerase (RdRp) protein tagging it for degradation, thus terminating viral genome replication [8]. Similarly, phosphorylation of Tomato yellow leaf curl virus (TYLCV) βC1 by host SnRK1 kinase results in its inactivation [9]. Another common and effective strategy involves ubiquitination of viral proteins for proteasome-mediated degradation. Due to their roles in the intracellular and intercellular movement, often exposing themselves to host factors, it is likely that movement proteins (MP) are optimal targets for host-mediated degradation [10]. For example, MP from Tobacco mosaic virus (TMV), Cotton leaf curl Multan virus (CLCuMuV βC1) and Tomato yellow leaf curl China virus (TYLCCNV βC1) are direct targets for host ubiquitination leading to their degradation [11–14]. It is proposed that viruses are masters in remodelling cellular systems for their exploitation, including the highjacking of the host PTM machinery for their counter-defence and infection. In agreement with this, many geminiviral proteins require PTMs for their activity as seen in Tomato leaf curl Yunnan virus (TLCYnV C4) and Cabbage leaf curl virus (CaLCuV NSP) proteins that require phosphorylation for their activity [15, 16].

Many studies link PTMs such as ubiquitination, phosphorylation and SUMOylation as major elements of the PTM-derived regulation of cellular homeostasis and defence [17]. Ubiquitination (Ub) is a versatile PTM with up to eight different kinds of poly-Ub linkages leading to distinctive outcomes of the substrates. For example, K48 poly-ubiquitination is a major signal for proteasomal degradation [18], whereas K63 poly-ubiquitin linkage mediates cellular processes such as localization, DNA repair and autophagy [19]. Phosphorylation of proteins on the other hand is an addition of a charged and hydrophilic phosphoryl group into the side chain of amino acids, possibly changing the structure and function of the target protein [20]. SUMOylation too plays diverse roles such as in DNA repair sensing, stress response, indirect tagging of proteins for degradation and altering the subcellular localization of various proteins [21]. It is a highly dynamic transient modification involving a covalent addition of small ubiquitin-like moiety onto lysine residues of the substrate via an isopeptide linkage. The transient nature of SUMOylation is due to the presence of enzymes called Sentrin-specific protein proteases (SENPs) that are necessary for the maturation of SUMO, as well as the removal of linked SUMO from substrates via cleavage of isopeptide bond [22].

The ATP-dependent conjugation of SUMO involves recognition of the C-terminal di-glycine motif (GG) of SUMO protein by SUMOylation enzyme E1 which is a heterodimer of SAE1/SAE2 in Arabidopsis (AOS1/UBA2 mammals). A thioester bond formation leads to the linkage of SUMO with E1 which is then transferred to the conjugating enzyme SCE1 (homolog of UBC9 in mammals). SCE1 in an E3-dependent or E3-independent manner transfers the SUMO moiety by a covalent isopeptide linkage to the lysine residue of the target protein having a consensus sequence ψ-K-X-E/D (where ‘ψ’ represents a hydrophobic amino acid and ‘X’ represents any amino acid) [23, 24]. In conjunction with SUMOylation, another non-covalent interaction occurs on the SUMO-interacting motifs (SIMs) of the same protein. Almost all the proteins known to undergo SUMOylation have SIM sites, highlighting the importance of SIM in the SUMOylation process [25]. Interestingly, all examples of non-consensus SUMOylation occurring on lysines without a consensus SUMO motif indicate the importance of a functional SIM motif for SUMOylation [26]. Mechanistically, SIM sites function either by docking the conjugation enzyme close to the SUMOylation site or by locally increasing the concentration of SUMO moieties close to the consensus lysine leading to efficient SUMOylation [27]. In addition to the effect on SUMOylation, SIM sites can act as docking sites for other SUMOylated proteins, increasing the repertoire of cellular interactions [25]. Unlike ubiquitination, whose conjugation process depends on multiple E2 enzymes, SUMOylation uses a single E2 in combination with multiple E3s. SUMOylation and SIM interaction both lead to variable outcomes, affecting protein localization, stability and interactions with other partner proteins.

SUMOylation being a versatile PTM is used by plants as regulators of major pathways. For example, SUMOylation of DNA repair proteins upon DNA damage acts as the trigger for the assembly of proteins on the damage site [28]. In plants, SUMOylation of cellular defence switches acts as triggers for antiviral defence [17]. SUMOylation mediated by E3 SUMO ligase SIZ1 has been implicated in a negative regulation of salicylic acid (SA)-based defence signalling thereby regulating expression of pathogenesis-related (PR) genes [29]. In addition, SUMOylation-mediated disruption of defence regulators is a crucial step during viral infection. Several RNA viral proteins undergo SUMOylation upon infection. For example, NiB protein of Turnip mosaic virus undergoes SUMOylation leading to relocalization of SUMO nuclear pool, causing inactivation of defence regulators such as NPR1 [30].

Geminiviral satellite DNA-coded βC1 is a small protein of approximately 13–15 kDa. It is an important viral protein that counters various host defence mechanisms as well as acting as a movement protein in monopartite begomoviruses. It is also a viral suppressor of RNA silencing (VSR) of host silencing defence machinery and is shown to bind different nucleic acids [31]. It has been well documented that TYLCCNV βC1 gets degraded in host cells [13]. However, it is not clear if this degradation is a host defence mechanism or a viral strategy to regulate the expression of this protein in the infected cells. The latter idea is supported by the evidence that a relatively higher expression of βC1 protein is toxic to cells [32]. In line with this, overexpression of βC1 and other movement-associated proteins cause developmental defects when expressed as transgenes. Further, CLCuMuB βC1 interacts with host autophagy machinery. Perturbing interaction of CLCuMuB βC1 with autophagy regulator led to premature death of host thereby reducing viral propagation. These studies suggest a possible co-evolution between compatible host and the virus, leading to late or dormant infection [14].

In this study, we elucidated novel PTMs on Synedrella yellow vein clearing virus (SyYVCV) pathogenicity determinant βC1 protein. We identified the roles of SUMOylation motifs and SIMs of βC1 during viral infection. Using in vitro and in vivo methods, we show that βC1 undergoes SUMOylation in plants. Furthermore, using NMR and in vivo studies, we show that βC1 SIMs are responsible for its degradation, while SUMOylation of different sites in βC1 leads to different outcomes deciding the fate of βC1 proteins. We also show a potential way by which geminiviral βC1 can interact and subdue host defence response mediated by SUMOylation. Our results indicate the presence of a novel mechanism operating during plant-virus interactions involving PTMs, leading to the fine balance between function and stability on the one hand or inactivity and degradation on the other.

## Results

### SyYVCV βC1, a geminiviral pathogenicity determinant, undergoes SUMOylation in host plants

**Fig. 1:**
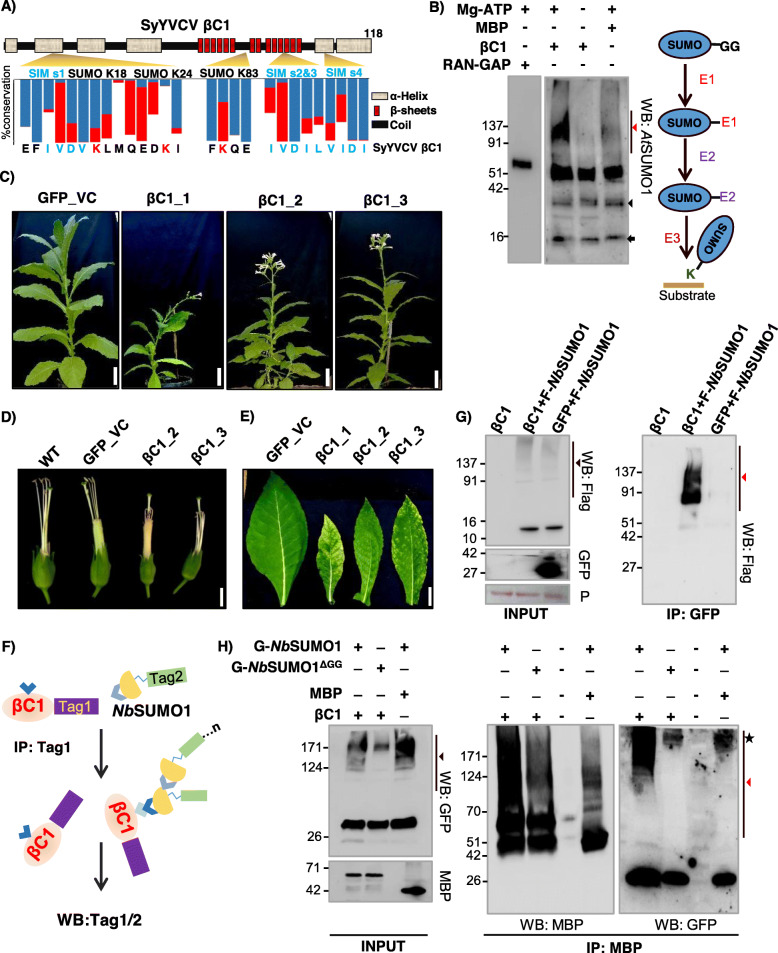
βC1 undergoes SUMOylation in vitro and in vivo. **a** Schematic showing predicted SUMOylation and SIM sites. Secondary structure was predicted using Predictprotein software. Blue bar indicates the percentage of amino acid conservation between βC1 sequences. The red bar shows the presence of structurally similar residue substitutions. **b** Left panel: in vitro SUMOylation with MBP-βC1 (59 kDa), MBP (42 kDa) and RanGap (63 kDa, positive control) using purified SUMO conjugating enzymes. Right panel: schematic of SUMOylation cascade. Red and black triangles represent poly-SUMOylated substrates and E1-SUMO conjugate, respectively. The black arrow indicates *Nb*SUMO1. **c** Phenotype of transgenic *N. tabacum* lines overexpressing eGFP-tagged βC1. **d** Flower phenotype (without petals). **e** Symptomatic leaves of transgenic βC1 plants. **f** Diagram showing the Co-IP presented in **g** and **h**. **g** Co**-**IP of transiently expressed GFP-βC1 (42 kDa) and vector GFP with co-expressed Flag-tagged *Nb*SUMO1 (F-*Nb*SUMO1). **h** Co-IP of transiently expressed MBP-βC1 (59 kDa) with co-expressed GFP-tagged *Nb*SUMO1 and *Nb*SUMO1^ΔGG^. Size bars in **c**, **d** and **e** are 10, 0.8 and 3 cm, respectively. Black and red triangles indicate *Nb*SUMO1 poly-SUMOylated proteins and *Nb*SUMO1-βC1 conjugates, respectively. The asterisk indicates a non-specific band. P, Ponceau staining showing RUBISCO large subunit. Protein sizes are shown in kDa

SyYVCV is a new monopartite Begomovirus recently characterized by our group, having a 2.7-kb DNA A component and a satellite DNA β of 1.3 kb length [33]. It causes vein clearing disease in its natural host and leaf curling in *Nicotiana tabacum*. SyYVCV βC1 is 118 aa long protein with multiple intrinsically disordered regions coded by the only ORF known in DNA β. Using bioinformatics tools (GPS SUMO, JASSA) [26, 34], we identified three putative SUMOylation sites spread throughout the length of the protein (Fig. 1a). Among the three predicted SUMOylation sites (Ss), Ss1 (Lysine, K18) and Ss2 (Lysine, K24) showed inverted SUMOylation consensus (D/EXKψ), whereas Ss3 (K83) was predicted to have a consensus site for SUMOylation (ψKXE/D) with a low score (Additional file 1: Fig. S1A). Both inverted and consensus sites usually get SUMOylated [35].

Among the three predicted sites, Ss1 (K18) was the most conserved SUMOylation site (77% of all βC1 entries from the nr Uniprot database) when compared between viruses that are associated with β DNA. This was followed by Ss3 (K83; 37%). Ss2 (K24) was least conserved among all predicted sites (10%), mostly restricted to SyYVCV and AYVV (Ageratum Yellow Vein Virus) cluster of viruses that result in vein clearing symptoms (Fig. 1a, Additional file 1: Fig. S1B and Additional file 2: Table S1).

To validate whether SyYVCV βC1 is a direct target for SUMO conjugation, an in vitro SUMOylation assay containing recombinant *E. coli*-purified MBP-βC1, SUMO-activating enzyme mixture (E1 homolog), Ubc9 (E2 homolog) and His-tagged SUMO1 (*Nb*SUMO1-GG, C-terminal activated SUMO1 *N. benthamiana*) was performed. We observed multiple slow-migrating high molecular weight intermediate products in a denaturing PAGE gel when blotted with anti-*At*SUMO1 antibody only in the presence of βC1 as substrate. These bands were detected only in the presence of Mg^2+^-ATP, and likely represented a SUMOylated βC1 (Fig. 1b). The SUMOylated products were a result of the direct reaction involving E1 and E2, since the absence of E1 and E2 in the reaction did not produce higher-order bands (Additional file 1: Fig. S2A). These results likely suggested the SUMOylation of βC1 in vitro in an E1, E2-catalysed ATP-dependent reaction. To further validate this interaction, we performed yeast two-hybrid (Y2H) assay with *Nb*SUMO1 as prey and βC1 as bait. We observed the growth of yeast cells, suggesting an interaction between *Nb*SUMO1 and βC1 (Additional file 1: Fig. S2B).

To understand the biological function of βC1 SUMOylation, we generated transgenic *N. tabacum* plants overexpressing βC1. As expected and observed in case of overexpression of many pathogenicity determinants, overexpression of βC1 induced symptoms similar to virus-infected plants [36] (Fig. 1c, d, e, and Additional file 1: Fig. S3). These plants exhibited abnormal phenotypes such as stunted growth, early flowering, pointed leaves, shorter internodes, branching and mosaic patches on the leaves. In addition to these phenotypes, the expression of βC1 induced exerted stigma phenotype and seed sterility. Although the expression of a DNA viral protein in plants was not previously reported to show exerted stigma phenotype, RNA viral proteins are known to produce such defects [37].

To substantiate the defects observed in transgenic plants overexpressing βC1 was because of the interactions mediated by βC1 SIM and SUMOylation motifs, we verified whether βC1 undergoes SUMOylation *in planta*, we performed co-immunoprecipitation (Co-IP) of βC1 from stable transgenic plants overexpressing βC1. βC1, but not control GFP expressing plants, showed high molecular weight bands when blotted with anti-GFP (Additional file 1: Fig. S4A) and anti-*At*SUMO1 antibodies (Additional file 1: Fig. S4B). Since SUMOylation is a dynamic process and less than 1% of any substrate protein is SUMOylated at a given point in cells [21], we transiently overexpressed 3X Flag-tagged *Nb*SUMO1 and GFP-tagged βC1 in *N. tabacum* to increase the chance of βC1 SUMOylation and its detection through WB (Fig. 1f). Co-IP of βC1 was performed, followed by western blot analysis with anti-FLAG antibody. Any signal from the pull-down products after blotting with anti-FLAG antibody essentially indicates an interaction of *Nb*SUMO1 with βC1 (Fig. 1g). After Co-IP with anti-GFP and detection with anti-FLAG antibody, we observed signals ranging from 60 to 150 kDa only in βC1 pull-down products, but not in control, indicating the presence of SUMO-conjugated βC1 (Fig. 1g). SUMOylation of βC1 produced higher-order intermediates likely due to SUMOylation of multiple SUMO conjugation sites of βC1 as well as poly-SUMOylation of *Nb*SUMO1 conjugated to βC1 [38].

We further validated these results by swapping tags and using a non-conjugable form of *Nb*SUMO1 (*Nb*SUMO1Δ^GG^) in the abovementioned assays. During the SUMOylation process, SUMO proteins undergo proteolysis at their C-terminus to expose their di-Glycine motifs. As a result, when we transiently overexpressed *Nb*SUMO1Δ^GG^ in plants, higher-order conjugation products were absent at the global level suggesting inefficiency of *Nb*SUMO1Δ^GG^ to undergo SUMOylation (Additional file 1: Fig. S4C). We used GFP-tagged *Nb*SUMO1 and *Nb*SUMO1Δ^GG^ to validate that the higher-order bands observed are actual conjugated products of *Nb*SUMO1 derived from the SUMOylation cascade. We used MBP-tagged βC1 as a substrate for detecting SUMOylation along with GFP-tagged *Nb*SUMO1in conjugable and non-conjugable forms. After Co-IP with anti-MBP followed by blotting with anti-GFP and anti-MBP, we observed high molecular weight bands in βC1 when co-expressed with a conjugable form of *Nb*SUMO1, but not with non-conjugable form (Fig. 1h).

*Nb*SUMO1 is the only identified SUMO protein in *Nicotiana* sp., whereas the model plant *Arabidopsis* has 4 characterized SUMO proteins [39]. To further explore the possibility of interaction between βC1 and other SUMO proteins, we used Y2H assay. As SUMO1 and SUMO2 have highly redundant biochemical functions in *Arabidopsis*, we used only SUMO1, SUMO3 and SUMO5 of *Arabidopsis* as prey proteins fused to activation domain (AD) in a yeast two-hybrid screen with βC1 fused to the binding domain (BD). We observed a strong interaction of βC1 with *At*SUMO3 and *At*SUMO5 (Additional file 1: Fig. S5A). *At*SUMO5 caused auto-activation when fused to AD domain alone; however, the strength of interaction with βC1 in the quadruple (-LWHA) knockout media was clearly observed. Auto-activation caused by *At*SUMO5 was minimal in -LWHA, but in the presence of βC1, the growth of cells was enhanced suggesting interaction. In the case of *At*SUMO3, a strong interaction was observed only with βC1, indicating βC1 might also interact with *At*SUMO3. To distinguish between SUMOylation and SIM-mediated interactions, we used di-Glycine deleted *At*SUMO3 and *At*SUMO5. Interestingly, the deletion of the di-Glycine motif caused no difference in the interaction of βC1 with *At*SUMO5, but completely abolished its interaction with *At*SUMO3. These results suggest that other SUMO proteins might also interact with βC1 via SUMOylation or SIM-mediated interactions. In these assays, protein expression and stability of all proteins were verified. All other proteins except *At*SUMO5 were expressing at almost equal levels in yeast cells, while *At*SUMO5 was expressed at unusually high levels, and might be the reason for its auto-activation (Additional file 1: Fig. S5B) [40]. To further verify the biological significance of the observed interaction between βC1 and other SUMO proteins, we overexpressed GFP-tagged versions of both *At*SUMO3 and *At*SUMO5 along with βC1 and performed an IP with βC1 as previously described (Fig. 1f). We observed a very weak pull-down signal of *At*SUMO3 and *At*SUMO5 as compared to *Nb*SUMO1 (Additional file 1: Fig. S5C). These experiments suggest that even though βC1 is able to interact with other SUMO proteins in yeast, in *Nicotiana* sp., βC1 majorly interacted with *Nb*SUMO1. Together, these results strongly indicate that βC1 undergoes SUMOylation in plants and that it interacts with host SUMO proteins.

### SUMOylation sites are essential for the stability of βC1 in host plants

**Fig. 2:**
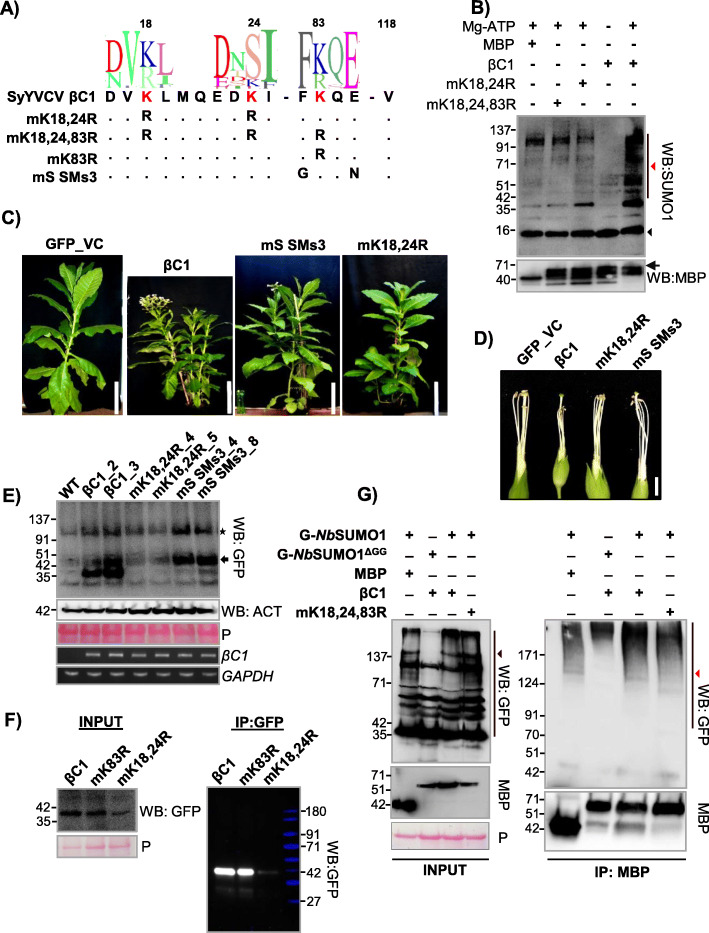
Mutations in βC1 SUMOylation sites abolish pathogenicity. **a** Design of βC1 SUMOylation motif mutants. SeqLogos indicates conservation in amino acid sequences between βC1. **b** In vitro SUMOylation assay using purified MBP-βC1 and its SUMOylation motif mutants. Top blot shows SUMOylation conjugates of βC1 probed with anti-SUMO1 and bottom blot is reprobing with anti-MBP. Black arrow indicates intact MBP-βC1. Black and red triangles represent NbSUMO1 and poly-SUMOylated substrates, respectively. **c**, **d** Representative phenotypes of GFP-βC1 and its SUMOylation motif mutants in transgenic *N. tabacum*. **c** Plants. **d** Flowers without petals. **e** Western blot to quantify GFP-βC1 and different SUMOylation motif mutants in transgenic plants using anti-GFP. The star indicates a non-specific band and the arrow shows GFP-βC1. **f** IP of GFP-βC1 and its SUMOylation motif mutants showing stability of the proteins during transient overexpression. Protein ladder overlaid and false colour applied. **g** Co-IP of MBP-βC1 and its SUMO mutants during co-overexpression of either *Nb*SUMO1 or *Nb*SUMO1^ΔGG^. Black and red triangles indicate *Nb*SUMO1 poly-SUMOylated proteins and *Nb*SUMO1-βC1 conjugates, respectively. Protein marker sizes in kDa are indicated. Size bars in **c** and **d** are 36 cm (1.2 ft) and 0.8 cm respectively. P, Ponceau staining for total proteins showing RUBISCO

Since there are three predicted SUMOylation sites in βC1 (Fig. 1a), we explored which among these predicted sites are necessary and sufficient for SUMOylation. We substituted lysine residues to arginine, which will abolish SUMO modification of the consensus sequence without leading to much structural disruption. Since Ss1 (K18) and Ss2 (K24) residues of βC1 are close to each other, we designed a double mutant K18, 24R (henceforth mK18, 24R) to cover both these sites, a single Ss3 (K83) mutant (mK83R) and a null mutant with all three predicted lysines mutated to arginines, i.e. K18R, K24R and K83R (mK18,24,83R) (Fig. 2a). We recombinantly expressed and purified the abovementioned mutants of βC1 from *E. coli* and performed an in vitro SUMOylation assay with *Nb*SUMO1. We observed that all predicted lysines (K18, 24 and 83) underwent *Nb*SUMO1 conjugation and mutating these sites to arginine in double mutant or in triple null mutant abolished *Nb*SUMO1 modification in vitro (Fig. 2b). To further confirm the above observations, we also performed an in vitro SUMOylation assay with *Nb*SUMO1 using short peptides covering the βC1 SUMOylation consensus lysine sites. Mutating SUMOylation sites inhibited *Nb*SUMO1 conjugation in vitro (Additional file 1: Fig. S6A). These results indicate that there is a propensity of all 3 predicted sites of βC1 to undergo SUMOylation in vitro.

To further understand the importance of these SUMOylation sites of βC1, we generated transgenic plants overexpressing SUMOylation site mutants of βC1. Along with mK18, 24R, a Ss3 mutant was generated where the consensus lysine K83 was kept intact, while SUMOylation consensus sequence was removed (mS SMs3, Fig. 2a). Interestingly, transgenic plants overexpressing mK18, 24R double mutant was devoid of abnormal phenotypes and was similar to GFP overexpressing control plants (Fig. 2c), indicating that SUMOylation in K18, K24 residues of SyYVCV βC1 is essential for the development of symptoms in plants. mS SMs3 mutant overexpressing transgenic plants also showed recovery from severe phenotype to some extent (Fig. 2d and Additional file 1: Fig. S6B, C and D).

**Fig. 3:**
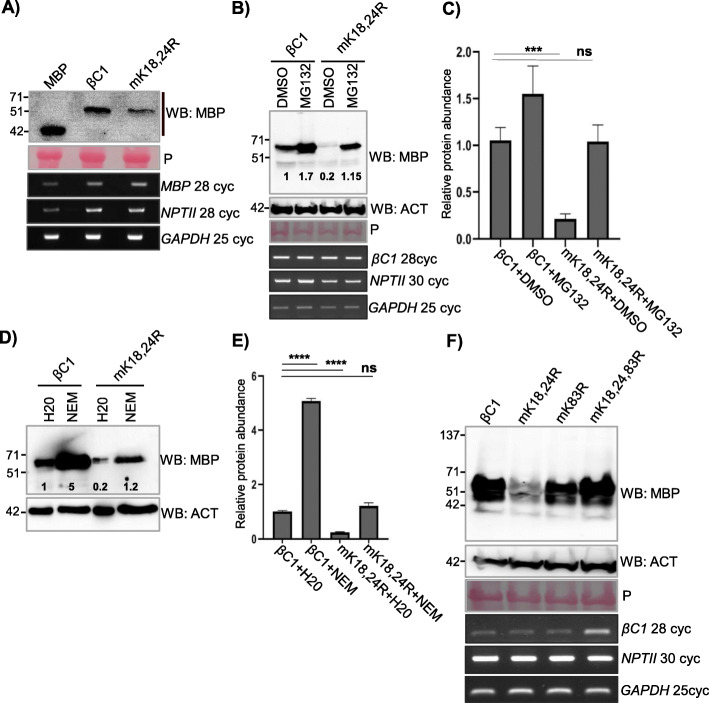
SUMOylation stabilizes βC1 protein in plants. **a** Stability of transiently overexpressed MBP-βC1 and mK18, 24R SUMOylation-deficient double mutant in *N. tabacum*. Samples were collected 3 DPI. **b** Accumulation of transiently overexpressed MBP-βC1 and mK18, 24R upon MG132 treatment. **c** Quantification of protein levels after MG132 treatment. *n* = 3. **d**, **e** Same as **b** and **c** but with NEM treatment. **f** Stability of transiently overexpressed MBP-tagged βC1 and its SUMOylation motif triple mutant. The number of PCR cycles (cyc) in RT-PCR experiments has been mentioned. Tukey’s multiple comparison test with four stars representing *P* value, *P* ≤ 0.0001 and three stars *P* ≤ 0.001. P, Ponceau staining for total proteins showing RUBISCO large subunit. Other information same as in Fig. 1

In order to understand why transgenic plants expressing mK18, 24R double mutant were asymptomatic, we performed detailed molecular analysis. Our immunoblot analysis of βC1 and SUMOylation-deficient mutant plants revealed a reduced protein accumulation of mK18, 24R double mutant as compared to WT βC1 or other mutants (Fig. 2e). SUMOylation-deficient double mutant mK18, 24R protein accumulated only 10% of WT βC1 (Fig. 3a and Additional file 1: Fig. S7A). mS SMs3 mutant protein levels were comparable to that of WT βC1(Fig. 2e). The transgenic plants expressing βC1 mutant showed disproportionate severity of symptoms, likely indicating a hierarchy in these sites to undergo PTMs and subsequent functions. Reduction in mK18, 24R protein level was not due to reduced transcription as seen in RT-PCR analysis (Fig. 2e). Further, to verify the observations of transgenic plants, we transiently overexpressed WT βC1, mK18, 24R and mK83R mutants in *N. tabacum* and performed an IP for βC1. Similar to transgenic plants, mK18, 24R mutant levels were significantly reduced in our IP analysis performed from transient overexpression of these proteins (mK18, 24R and mK83R) (Fig. 2f). To understand the SUMOylation status of these three lysines of βC1, we performed a Co-IP assay by co-expressing MBP-tagged βC1 or mK18, 24, 83 R along with GFP-*Nb*SUMO1. We observed a significant decrease in SUMOylation of mK18, 24, 83R triple mutant, indicating that these sites are the major sites of *Nb*SUMO1 conjugation (Fig. 2g).

### N-terminal SUMOylation-deficient double mutant of βC1 is prone to enhanced degradation in plants

It was shown previously that geminiviral βC1 from diverse viruses undergo degradation in host cells. TYLCV βC1 undergoes ubiquitin-mediated proteasomal degradation, while CLCuMuV βC1 directly interacts with ATG8 which leads to an autophagy-mediated degradation [13, 14]. To check if mK18, 24R mutant of βC1 undergoes active degradation in the host, a time course protein analysis of βC1 and mK18, 24R mutant was performed (Additional file 1: Fig. S7B). SyYVCV βC1 protein levels were slightly reduced after 1 day post infiltration (DPI), whereas mK18, 24R accumulated only to half the level of βC1 at 1 DPI and was barely detectable at 2 DPI. To understand the cause of reduced accumulation associated with SUMOylation-deficient mK18, 24R mutant, we employed degradation pathway inhibitors to check if mK18, 24R SUMOylation-deficient mutant undergoes enhanced degradation in vivo. Upon treatment with MG132 (carbobenzoxy-l-leucyl-l-leucyl-l-leucinal), a potent reversible inhibitor of proteasomal activity, protein levels of βC1 increased substantially (Fig. 3b), indicating that it undergoes active proteasomal degradation in plants. Interestingly, we also observed a drastic increase in the levels of mK18, 24R mutant whose protein level stabilized more than WT βC1 upon MG132 treatment (Fig. 3b and c quantification). We further used a wide spectrum protease inhibitor *N-ethylmalemide* (NEM) that inhibits cysteine proteases and partially inhibits proteasome [41]. Upon treatment with NEM, mK18, 24R mutant protein levels increased 3- to 5-fold (Fig. 3d and e quantification), suggesting enhanced degradation of mK18,24R βC1 mutant in plants. These assays suggest that the N-terminal SUMOylation-deficient mutant of βC1 undergoes enhanced degradation mediated by the host protein degradation pathway.

As observed in protein expression analysis from transgenic plants expressing SUMOylation mutants of βC1, there appears to be a disparity between three SUMOylation motifs of βC1. To further understand the functional significance of these N- and C-terminal localized SUMOylation motifs of βC1, we performed transient overexpression assays for the abundance of mK18, 24R, mK83R and mK18,24, 83 R βC1 mutants. While the mK18, 24R mutant was unstable in transient assay as well as in transgenic plants, a triple SUMOylation site mutant (mK18, 24, 83R) was surprisingly stable (Fig. 3f). To pinpoint the exact SUMOylation motif involved in the stability of βC1, we generated individual K to R mutants of N-terminal SUMOylation motifs K18 and K24. Interestingly, none of the single mutants was unstable (Additional file 1: Fig. S7C). We speculated that removing all three SUMOylation sites either altered recognition of the protein or other steps in proteasome-mediated degradation, leading to the stability of the protein as may be the case of mK18, 24, 83R. To further confirm that the enhanced degradation of SUMOylation-deficient mK18, 24R mutant in plants is via the plant degradation pathway and not due to intrinsic instability of mutants, we expressed βC1, mK18, 24R and mK18, 24, 83R in a yeast WT strain (*BY*4741). All the mutants were as stable to levels comparable to WT βC1 protein (Additional file 1: Fig. S7D). Taken together, all these results confirm that SyYVCV βC1 undergoes rapid degradation in plants similar to other viral βC1 proteins observed previously. These results also indicate that due to loss of protective marks, mK18, K24R degradation is enhanced suggesting the significance of N-terminal SUMOylation motifs in the stability of βC1.

### SUMO-interacting motifs (SIMs) of SyYVCV βC1 interact with *Nb*SUMO1

In multiple proteins that undergo SUMOylation, a complementary stretch of SIM was routinely observed within the candidate protein. In SyYVCV βC1, along with three SUMOylation motifs, four SIMs were predicted using SIM prediction softwares JASSA and GPS SUMO. The N-terminal SIM (residue 14–17, SIM1) overlaps with the K18 SUMOylation motif consensus sequence. The second and the third predicted SIM sequences are in an overlapping stretch forming SIM2, 3 (residue 90–93 and 91–94) (Additional file 1: Fig. S8A). The last SIM (SIM4) (residue 101–104) is towards the extreme C-terminal end. It is important to note that SIM2, 3 and SIM4 are in close proximity to the third SUMOylation motif consensus lysine K83.

**Fig. 4:**
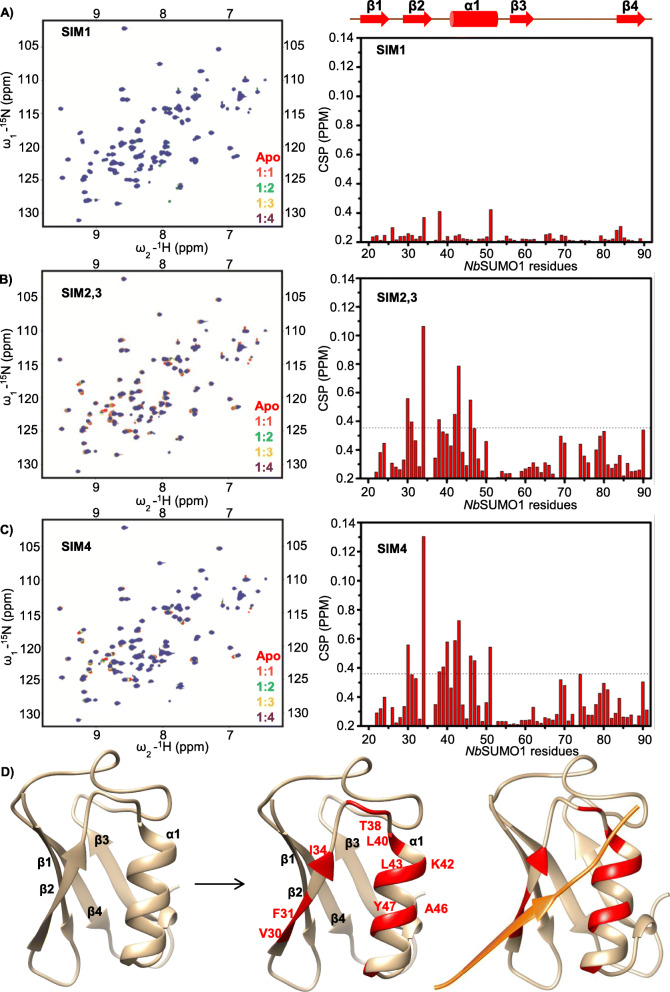
βC1 SIMs interact with *Nb*SUMO1 as seen with ^15^N-^1^H HSQC spectrum. **a** SIM1, **b** SIM2,3 and **c** SIM4 (left panel). The right panel shows corresponding CSPs between free and *Nb*SUMO1-SIM-bound form plotted against individual residues of *Nb*SUMO1. The dashed line indicates mean ± SD of CSP values. Residues above the dash line show binding interface. **d** Structure of *Nb*SUMO1. Left panel: *Nb*SUMO1 predicted structure, ribbon and surface representation. Middle panel: residues of *Nb*SUMO1 interacting with βC1 SIM motifs (Red). Left panel: *Nb*SUMO1/βC1-SIM4 model. SIM4 is shown in orange

Plant SUMO proteins are diverse and form a distinct clade even though the SUMO proteins are highly conserved from yeast to mammals (Additional file 1: Fig. S8B). In *Arabidopsis*, eight SUMO coding genes are known, out of which four SUMO proteins (*At*SUMO1, 2, 3 and 5) are known to express and being observed to be functionally active. As reported earlier, we observed differential tissue-specific expression of *Arabidopsis* SUMO proteins (Additional file 1: Fig. S9A). To identify the SUMO proteins potentially interacting with βC1 SIMs, we used NMR titration experiments. We recombinantly expressed and purified ^15^N-labelled SUMO 1, 2, 3 and 5 from *Arabidopsis*, and SUMO1 from *N. benthamiana* (Additional file 1: Fig. S9B). Interestingly, *At*SUMO3 and 5 were insoluble in *E. coli* (Additional file 1: Fig. S9C) and, after refolding, exist as soluble higher-order multimers (Additional file 1: Fig. S9G and S9H) whereas *Nb*SUMO1 and *At*SUMO1 exist as monomers (Additional file 1: Fig. S9D, E and F). The physiological significance of this multimerization property of plant SUMO proteins is unknown; however, many studies have observed *At*SUMO3 and *At*SUMO5 localized as nuclear speckles [30]. *Nb*SUMO1 and *At*SUMO1 are structurally identical with a pairwise sequence identity of 97%. We used *Nb*SUMO1 for screening multiple SIMs of SyYVCV βC1 through ^15^N-edited Heteronuclear Single Quantum Coherence (HSQC) experiments using SIM peptides (Additional file 1: Fig. S10A). Upon titration with the SIMs derived from βC1, *Nb*SUMO1 showed interaction with SIM2, 3 and SIM4 (Fig. 4b, c, left panel). However, SIM1 did not show any interaction with *Nb*SUMO1 (Fig. 4a, left panel). Based on the NMR analysis, a structural model of *Nb*SUMO1 indicating the residues involved in interaction with βC1 SIMs were predicted (Fig. 4d). The chemical shifts of the backbone ^1^HN, ^15^N, ^13^Cα, ^13^Cβ and ^13^CO resonances of the *Nb*SUMO1 were assigned by standard triple resonance NMR experiments (see the “Methods” section) (Additional file 1: Fig. S11A). The chemical shifts were used in a modelling software CS-ROSETTA [42] to obtain a structural model of *Nb*SUMO1 (Fig. 4d, right panel). The chemical shift perturbations (CSPs) of SIMs were mapped on the *Nb*SUMO1 structure to highlight the SUMO: SIM interface (Fig. 4d, middle panel). To understand the structural interaction and binding pocket involved in *Nb*SUMO1: βC1 SIM interaction, we modelled them together based on the CSP data and human SUMO1/IE2-SIM structure (PDB id: 6K5T) in UCSF-Chimera (Fig. 4d, left panel). IE2 is a human cytomegalovirus protein [43]. The CSPs identified residues 30 to 50 as the region of *Nb*SUMO1 binding βC1 SIM 2, 3 motif (Fig. 4b, right panel) and SIM 4motif (Fig. 4c, right panel and Additional file 1: Fig. S11B). To validate these interactions, we further used SIM mutants in our HSQC experiments. As expected, SIM2, 3 or SIM4 mutants did not interact with *Nb*SUMO1 (Additional file 1: Fig. S11C, D and S11E).

We also verified *Nb*SUMO1 interaction with βC1 using in vivo and in vitro pull-down assays. βC1 was able to pull-down GFP-tagged *Nb*SUMO1 from plants as well as recombinantly purified *Nb*SUMO1 in an in vitro pull-down assay. We purified SIM mutated βC1 proteins and used them as bait to pull-down *Nb*SUMO1 (recombinantly expressed as 6X HIS *Nb*SUMO1 in *E. coli* or transiently expressed in plants as GFP-*Nb*SUMO1). As SIM2, 3 and SIM 4 can separately bind to *Nb*SUMO1, mutating both SIM 2, 3 and SIM4 abolished non-covalent interactions with SyYVCV βC1 (Additional file 1: Fig. S12A and S12B).

### SIMs of SyYVCV βC1 are essential for its function as a symptom determinant

**Fig. 5:**
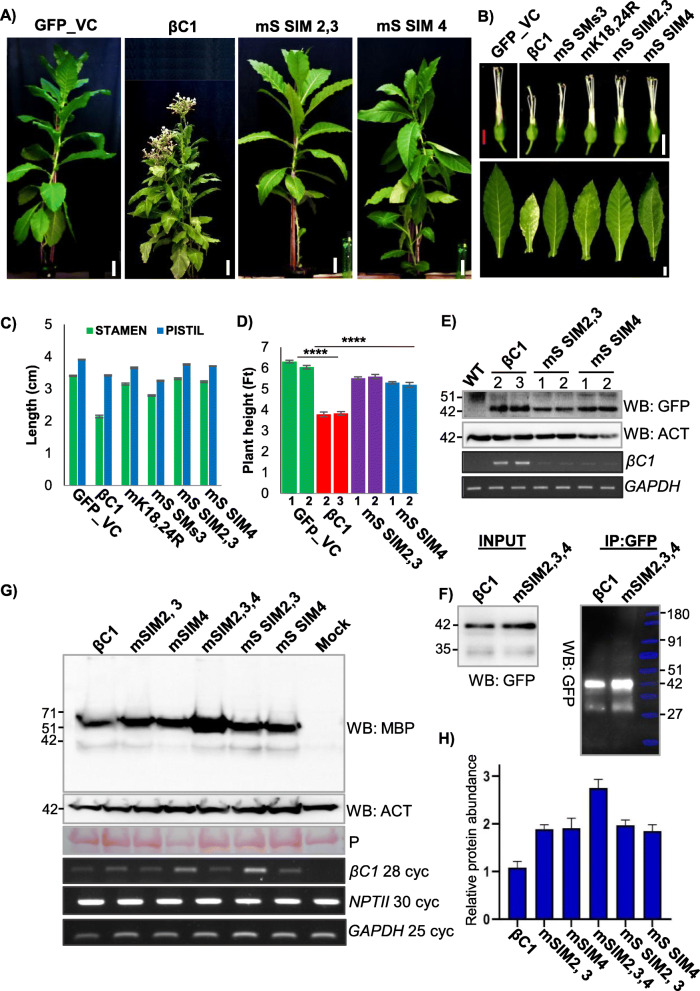
Mutations in SIM abolished infectivity of βC1. **a**–**d** Habit, flower, leaf and height of transgenic GFP-βC1 and its SIM mutants in *N. tabacum* transgenic lines (*n* = 3). Photographs and measurements (graphs) are presented. **e** Stability of βC1 and SIM mutants overexpressing N-terminal GFP-tagged proteins in transgenic plants. Numbers denote individual transgenic lines. **f** Stability of SIM mutants during transient overexpression. IP of N-terminal GFP-tagged βC1 and mSIM2,3,4 mutants derived from transient overexpression in *N. tabacum*. **g** Western blot showing protein levels during transient overexpression of MBP-tagged βC1 and SIM mutants (structural and null mutants) in *N. tabacum.* **h** Quantification of **g**, the *y*-axis represents protein expression in arbitrary number. *n* = 3. Marker sizes in kDa are indicated. Size bar in **a** 10 cm, **b** top-panel 0.8 cm and bottom panel 2 cm. P, Ponceau staining for total proteins showing RUBISCO large subunit

SIMs play an important role in protein-protein interactions. To gain further insight into the functional significance of SIMs in βC1, we generated *N. tabacum* transgenic lines expressing βC1 mutants, where SIMs were mutated to structurally similar motif but without the potential to interact with SUMO (Additional file 1: Fig. S10B). The single mutant of either SIM2, 3 (mS SIM2, 3) or SIM4 (mS SIM4) reverted most of the symptoms observed in βC1 overexpressing transgenic lines (Fig. 5a). Although single SIM mutants of βC1 transgenic plants had reduced severity of the symptoms when compared to WT βC1, they still exhibited mild symptoms such as yellowing of leaves and enhanced branching (Fig. 5b). However, unlike WT βC1, transgenic lines stably expressing βC1 SIM mutants were fertile, with no defect in floral organs and produced viable seeds (Fig. 5b–d). To understand the basis for this phenotype reversal, we further quantified the level of protein expression of the SIM mutants in transgenic plants. Unlike mK18, 24 R mutant, single SIM mutants were stable in plants and maintained protein levels comparable to that of WT βC1 (Fig. 5e). We further validated the stability of SIM mutants by transiently overexpressing C-terminal SIM mutant proteins in plants followed by an IP analysis (Fig. 5f). To reinforce our observation, we transiently overexpressed βC1 with its SIM site mutated to structurally similar motif or to an alanine patch completely removing SIM potential (Fig. 5g and h quantification) (Additional file 1: Fig. S10B). Surprisingly, SIM mutants were much more stable than WT βC1, and mutating a single C-terminal SIM motif was sufficient to increase the stability of the protein considerably. These results suggest that SyYVCV βC1 SIM motifs also play an important role in its function and stability.

Since removing SIMs of βC1 led to an increase in protein stability, we mutated SIM 2, 3 and SIM 4 of βC1 N-terminal SUMOylation motif mutant (mK18, 24R). The N-terminal SUMOylation motif mutant (mK18, 24R) had reduced stability and underwent rapid degradation, whereas upon additional mutations in SIMs enhanced the stability of the protein (Additional file 1: Fig. S12C). We further performed a time course experiment to check for the stability of these SIM and SUMOylation motif mutants of βC1. Unlike SUMOylation motif mutant (mK18, 24R), protein levels of SIM mutated βC1 were much higher at both 1 and 3 DPI even more than WT βC1 levels (Additional file 1: Fig. S12D and 12E quantification).

To understand the reason behind increased stability of SIM mutants, we performed a pull-down experiment using C-terminal SIM mutant (mSIM2, 3, 4) and screened for poly-ubiquitination. WT βC1 exhibited poly-ubiquitination as expected and in accordance with previous studies [12, 13, 44]. MBP control showed very little poly-ubiquitination signal. Very interestingly, SIM mutant unlike WT βC1 did not accumulate poly-ubiquitin chains (Additional file 1: Fig. 12F), indicating that disruption of SIM sites is necessary and sufficient to block ubiquitination. Global ubiquitination was not reduced in any of these samples (Additional file 1: Fig. 12F, Input).

### SUMOylation motifs and SIMs of SyYVCV βC1 are necessary for its function as a viral counter-defence protein

**Fig. 6:**
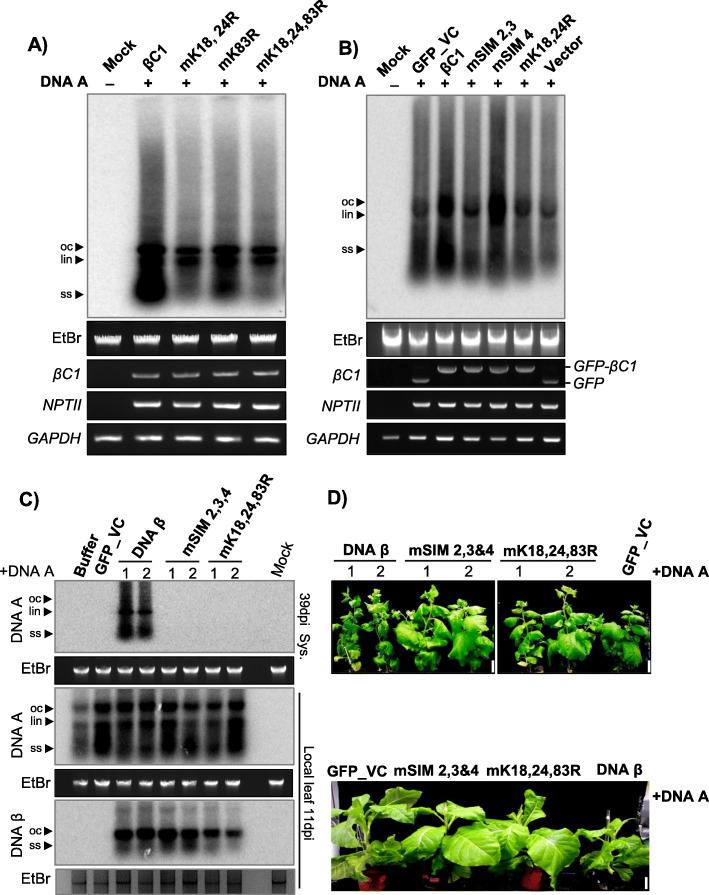
SUMO/SIM mutants of βC1 block viral systemic movement. **a** Southern blot viral replication assay in local leaves of transgenic plants expressing p35S::GFP-βC1 or its SUMOylation motif mutants. Infectious DNA A clones were used for infection. Total genomic DNA was treated with S1 nuclease before blotting. **b** Viral replication assay using SIM mutants of GFP-βC1. **c** Southern blot showing viral replication and systemic movement with DNA β and SIM/SUMO mutated βC1 variants incorporated in DNA β in local and systemic leaves of *N. benthamiana.* **d** Phenotypes of SyYVCV + DNA β or SIM/SUMO mutated βC1 incorporated in DNA β in *N. benthamiana* (top) and *N. tabacum* (bottom). Images were taken at 39 DPI. Size bar 5 cm

βC1 from multiple begomoviruses have been shown to act as host defence suppressors and mediators of viral replication in host plants. Local viral replication assay was performed to ascertain the role of βC1 in augmenting viral replication. Co-inoculation of infectious SyYVCV DNA-A partial dimer along with p35S: SyYVCV βC1 in *N. tabacum* leaves led to enhanced viral accumulation in local leaves (Additional file 1: Fig. S13A). However, mutating SUMOylation motifs of SyYVCV βC1 led to the complete abolishment of βC1 activity thereby decreasing the viral accumulation (Fig. 6a). Substitution of p35S: SyYVCV βC1 with SIM mutants also reduced viral replication in case of SIM2, 3 mutant (Fig. 6b). Similar results were obtained while infecting SyYVCV DNA-A partial dimer on SIM or SUMOylation motif mutant overexpressing transgenic plants (Additional file 1: Fig. S13A and B). We further performed viral replication assay with individual SIM mutants, and as expected, null mutants and structural mutants behaved similarly. Substitution of WT βC1 with p35S: mSIM 2, 3, 4 triple mutant in local viral replication assay resulted in the loss of βC1 function as a viral replication augmenting protein, resulting in much reduced viral accumulation (Additional file 1: Fig. S13C).

However, surprisingly, when WT βC1 was substituted with its SIM4 mutant, we observed enhanced viral accumulation (Fig. 6b). The exact mechanism behind this local enrichment of virus upon mutating βC1 SIM 4 is not clear. Most probably, differential functions of the two validated SIMs played a role in this disparity observed in viral replication assay. SIM4 of βC1 binds to *Nb*SUMO1 with greater affinity than SIM2, 3 (Fig. 4c, right panel). Even transgenic lines expressing mutated SIM2, 3 or SIM4 motif showed distinct recovery phenotype as compared to WT βC1. Altogether, these results suggest that βC1 SIM and SUMOylation motifs are necessary for its function as a viral pathogenicity determinant protein.

### SUMOylation motifs and SIMs of SyYVCV βC1 are also essential for systemic viral movement

In bipartite begomovirus with DNA-A and DNA-B, the B component codes for ΒC1 and BV1 that act as movement-associated proteins in the systemic spread of the virus. In case of monopartite viruses with a β satellite, the function of ΒC1 and BV1 is fulfilled by β satellite that codes for a single βC1 protein. To verify the function of SyYVCV βC1 as a viral movement protein (MP) and to determine the importance of its SIM and SUMOylation motifs in viral movement, we co-inoculated partial dimers of SyYVCV DNA-A along with SyYVCV DNA-β with its only ORF coding for WT βC1 or mSIM 2, 3, 4 (SIM) or mK18, 24, 83R (SUMO) motif mutants incorporated in viral genome, in 3-week-old *N. benthamiana* plants. As replication of DNA β is assisted by DNA-A coded Rep protein, we first verified that all DNA β dimers are able to replicate locally irrespective of their mutation in βC1 SIM or SUMOylation motifs (Fig. 6c). We observed classic viral symptom development (slight yellowing and curling of newly emerging leaves) only in plants inoculated with DNA-β coding for WT βC1 (Fig. 6d, DNA β plants 1 and 2). Upon Southern analysis to verify viral replication in newly emerging systemically infected leaves, we observed the presence of DNA-A replicative form (RF) in the presence of WT βC1 containing DNA-β (Fig. 6c). The symptoms in plants co-inoculated with DNA-A and DNA β were much prominent at 39 DPI which was also verified by the increased accumulation of DNA-A in systemic leaves. However, we did not observe detectable levels of DNA-A RFs in plants inoculated with DNA-β carrying mutated βC1 of either SIM2, 3, 4, or that of SUMOylation motifs such as SUMO K18,24,83R (Fig. 6c, top blot and Additional file 1: Fig. S13D) even at an earlier time point. These results clearly suggest the importance of SIM and SUMOylation motifs of βC1 in modulating systemic infection by facilitating viral movement.

### SUMOylation of βC1 also affects its cellular localization

**Fig. 7:**
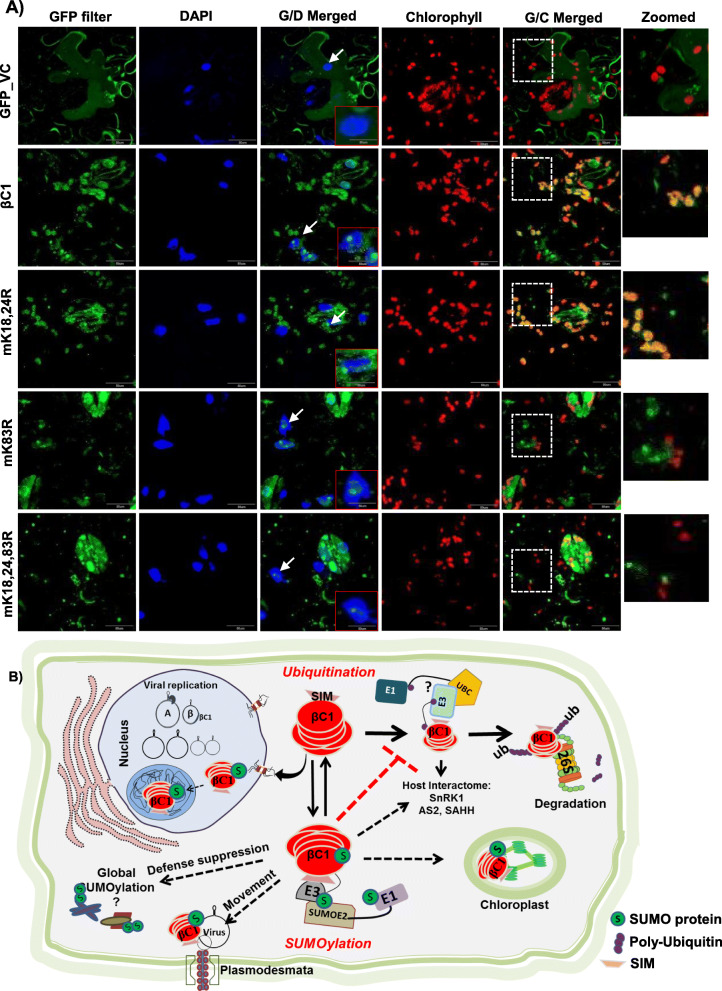
Mutations in SUMOylation motif of βC1 alters its localization. **a** GFP-tagged βC1 and its SUMOylation motif mutants were introduced onto *N*. *benthamiana* leaves as described in the “Methods” section. The nucleus was stained with DAPI, and chlorophyll auto-fluorescence was measured at 650 nm. βC1 localization in the nucleus and nucleolus is shown with a white arrow and a representative nucleus was zoomed in the red box. The white box with dashed line represents the zoomed area from G/C merged panel. G/D merged, GFP and DAP filter merged; G/C, GFP and chlorophyll auto-fluorescence merged. Size bar 50 μM. **b** Schematic representation showing interplay of multiple PTMs regulating function of βC1*.* Host ubiquitination machinery recognizes βC1 and an unknown ubiquitin ligase ubiquitinates βC1 via interaction with its SIM motif leading to its degradation. This inhibits viral movement. Viral counter-defence is achieved by interaction of βC1 with host SUMOylation machinery. SUMOylated βC1 is stable, and this aids in the movement of the virus and pathogenicity

SUMOylation of specific proteins has been implicated in altering the intracellular localization. In order to explore this possibility and to identify mechanism for the perturbations in the functions of βC1 upon SIM and SUMOylation motif mutations, we transiently expressed GFP-tagged βC1 and its mutants in epidermal cells of *N. benthamiana* leaves and analysed their localization using confocal microscopy. WT βC1 was diffusely localized in the nucleus and prominently in the nucleolus matching previous observations for related homologs [45]. Surprisingly, we also observed βC1 localizing in the chloroplast, strongly overlapping with chlorophyll auto-fluorescence shown in red (Fig. 7a, 2nd row). Mutating N-terminal double SUMOylation motif mK18, 24R did not result in any qualitative defect in chloroplastic localization (Fig. 7a, 3rd row). However, interestingly, we observed that the Ss3 mutant mK83R showed defects in chloroplastic localization even though its nucleolar localization was unaffected (Fig. 7a, 4th row). The same was observed in the case of triple SUMOylation motif mutant mK18, 24, 83R, where chloroplastic localization was significantly reduced (Fig. 7a, 5th row). We hypothesize that altered localization of βC1 mutants might have abolished its ability to support movement and replication of SyYVCV. In contrast, mutating SIM of βC1 did not alter the localization. SIM mutants were localized similar to WT βC1 in the nucleus, nucleolus and chloroplast, indicating SIM-mediated interactions did not affect localization (Additional file 1: Fig. S14A).

## Discussion

Cells use PTMs as a flexible way of reversibly and effectually controlling protein machinery. PTMs may act as proviral or antiviral machinery, and it has been shown that viruses infecting a range of hosts from yeast to mammals hijack this machinery during infection [46, 47]. Viral proteins might also interact with PTM machinery to regulate both proviral and antiviral effects. Viral proteins also routinely undergo PTMs to modulate infection. Recently, many geminiviral proteins (βC1, Rep, AC4, AV1) have been shown to undergo PTMs directly [9, 47–49]. We observed SyYVCV βC1 has multiple predicted SUMOylation consensus motifs as well as SIMs (Fig. 1a). This was intriguing since no other SyYVCV viral protein, except Rep, has any SIM or SUMOylation motifs. Although βC1 from other geminiviruses are modified by phosphorylation and ubiquitination, the functional significance of those PTMs is also not fully understood [9, 13]. In this report, we identified that SyYVCV βC1 gets SUMOylated in host cells to regulate its stability and to mediate interaction with cellular partners.

In few βC1 sequences, the SUMOylation consensus lysine residue was replaced by structurally similar arginine residue in Ss1 (K18) and Ss3 (K83) sites corresponding to SyYVCV βC1. However, since lysine is an absolute requirement for SUMOylation, arginine is unlikely to compensate. Such lysine to arginine replacements are commonly observed among a few viruses infecting tomato (TYLCV, TLCV and ToLCJV), cotton (CLCuV) and chilli (ChiLCV) complexes. Since substitution of lysine with arginine can inhibit SUMOylation even though other amino acids in the vicinity are conserved, this indicates the requirement of SUMOylation as a stronger selection than structural necessity (Fig. 1a and Additional file 1: Fig. S1B). We showed that SyYVCV βC1 undergoes SUMO conjugation by *Nb*SUMO1 in plants (Fig. 1g, h). We observed that upon mutating N-terminal SUMOylation site of βC1, there was a reduction in stability of βC1 (Fig. 2e).

SUMOylation can directly regulate the stability of the protein by inhibiting or increasing access to other PTMs, leading to multiple other functions including protein degradation (SUMO-targeted ubiquitin ligases). SUMOylation can also change the sub-compartmental localization of the protein to cut off access to degradation machinery. βC1 from TYLCCNV and CLCuMuV were previously shown to undergo active degradation in cells [12, 13]. We reasoned that the cause of reduced accumulation of N-terminal SUMOylation-deficient mutant of βC1 could be the enhanced degradation by cellular degradation machinery. In agreement with this, upon inhibition of protein degradation pathways, cellular levels of mutant βC1 (mK18, 24R) reverted to the same level or more than that of WT βC1 (Fig. 3b). As expected, this unstable mutant protein did not act as a pathogenicity determinant.

Almost all the proteins having a SUMOylation motif are accompanied by SIMs to play diverse functions in protein-protein interactions [25]. Although precise reasons for propensity of both these modifications in a single protein are still unclear, SIMs are known to help in SUMOylation by increasing the local concentration of SUMO proteins near the SUMOylation site(s). Moreover, SIMs have been shown to interact with SUMOylated proteins by increasing the repertoire of the interaction of a protein without having structural similarity [25]. Among the three SIMs in SyYVCV βC1, HSQC NMR analysis identified two SIMs located towards the C-terminus as mediators of interaction with *Nb*SUMO1 (Fig. 4b, c). Mutations in these two SIM motifs led to partial recovery from drastic phenotypes in transgenic plants. Surprisingly, unlike N-terminal SUMOylation motif mutants (mK18, K24R), SIM mutants exhibited enhanced stability of the mutant protein. Also, mutating individual or all SIMs led to increased stability of the protein in cells, suggesting partial redundancy of SIMs in function (Fig. 5g). These results indicate that N-terminal SUMOylation acts as a stability mark, whereas the C-terminal SIM interactions mediate degradation of βC1. Multiple observations are in agreement with this hypothesis, for example, mutating SUMOylation motifs removed the protective mark leading to increased degradation. On the other hand, the removal of SIM motifs led to stability. It is likely that SIMs act as marks for host-induced protein degradation that might be mediated by another SUMOylated protein or a protein that binds directly to the SIM patch. Intriguingly, mutating SIMs in N-terminal SUMOylation-deficient mutant background led to increased stability of the otherwise degradation-prone mutant protein (Additional file 1: Fig. S12C).

Interestingly, Haxim et al. [14] also reported a similar observation in CLCuMuV βC1. Here, V32 residue interacts with ATG8 autophagy mediator leading to βC1 degradation, whereas mutating V32 to A32 led to hyperactive protein and enhanced symptom induction during infection. In SyYVCV βC1, V32 residue has been replaced with a hydrophobic residue F32 that may likely abolish its interaction with ATG8. We hypothesized SyYVCV βC1 with only 33% pairwise sequence similarity with CLCuMuB βC1 may follow a similar but divergent strategy when compared to CLCuMuV during infection. Upon mutating SIMs, SyYVCV βC1 became ultrastable but lost its function as viral movement protein (Fig. 6c) and pathogenicity (no symptoms observed in transgenic plants expressing SIM mutated βC1 when compared to WT βC1) (Fig. 5a, b). This is in contrast to CLCuMuV βC1 where V32 residue was only necessary for protein turnover but not activity.

The C-terminal SUMOylation motif (K83) is in close proximity to SIM motifs (91 to 94 and 101-104 aa). Mutating either SIM motifs or the C-terminal K83 SUMOylation site enhanced the stability of the protein, suggesting a non-redundant but connected function in maintaining stability. Further, we observed that both the SIMs are necessary for function as mutating them led to the loss of symptoms in plants even though the proteins accumulated at optimal levels (Fig. 5a).

In par with our hypothesis, we observed a significant reduction in ubiquitination of C-terminal SIM mutant βC1 as compared to WT βC1 (Additional file 1: Fig. S12F). The exact molecular mechanism behind this reduction in ubiquitination is beyond the scope of this work; however, it is highly possible that mutating SIM motifs have deleted a recognition sequence of an unknown ubiquitin ligase, and as a result, it is unable to interact with βC1 causing a reduction in ubiquitination. Another possibility is the structural perturbation upon SUMOylation, from the example of PCNA (proliferating cell nuclear antigen), it is clear that both ubiquitination and SUMOylation are required for its function. In PCNA, ubiquitin binding and SUMO binding induced a distinct conformation [50]. It is highly likely that such a mechanism might be regulating βC1 protein, SUMOylation of βC1 protein leading to reversible modification of its structure, blocking accessibility of the ubiquitin ligases to βC1. Understanding structural perturbation caused by a PTM on a small but complex protein like βC1 is difficult due to the nature of the protein that forms heterogeneous soluble higher-order aggregates making it extremely troublesome to resolve even with cryo-EM (data not shown). We also cannot rule out another possibility where βC1 interacts with specific de-ubiquitinases as observed in HCV NS5A protein [51].

βC1 from various viruses have been shown to act as pathogenicity determinants, usually by enhancing viral replication. The function of βC1 as a movement protein was also impaired upon mutating SIM and SUMOylation motifs as observed in our viral replication assays (Fig. 6c). This loss of MP activity was not due to its inefficiency to bind ssDNA (Additional file 1: Fig. S15A). We hypothesize that as SIM and SUMOylation motifs are necessary for cellular interactions, mutations might have led to the loss of activity at the cost of its cellular interactions.

Manipulating SUMOylation pathway can be accepted as an important step in viral infection. Viral proteins modify multiple SUMOylation responsive proteins during infection [17, 52]. Viral manipulation of host SUMOylation machinery might lead to global change in SUMO conjugation as reported with adenovirus GAM1 protein [53] and Epstein-Barr virus (EBV) BRLF1 [54]. None of the plant viral proteins has been shown to have an effect on global SUMOylation. Many plant viral proteins like geminiviral Rep [55] and TuMV NIB [30] have been shown to interact and modify SUMOylation machinery. These viral proteins have specific targets and have not been implicated in global SUMOylation nor do they undergo SUMOylation. Our assays clearly show that SyYVCV βC1 induces an increase in global SUMOylation. This modification of global SUMOylation by βC1 is dependent on its SIM and SUMOylation motifs, since mutating either of the motifs compromised βC1 global SUMOylation modulating ability observed during both transient and transgenic stable expression of βC1 (Additional file 1: Fig. S15B, 15C and 15D). Perturbation in SUMO conjugation by mutating global SUMOylation regulators like SIZ1 (SUMO E3 ligases) or SUMO proteases (EDS4) led to global downregulation or upregulation of SUMO conjugation, respectively, producing early flowering and short stature phenotype in Arabidopsis [56, 57]. Same was observed in βC1 transgenic plants that flowered early and had stunted growth. These findings strongly point towards the manipulation of host SUMO conjugation machinery by βC1 during infection. Our findings that SyYVCV βC1 induce SUMO1-dependent global SUMOylation suggest that the cellular outcome of βC1 protein might be more extensive than is currently known. Further, SUMOylation of βC1 may also play a role in the already known functions of this protein.

We are also able to find additional host signatures altered due to global SUMOylation. It is well known that SUMOylation plays a crucial role in regulating the basal defence of host against pathogens mediated by PR genes [29]. It was shown that in SIZ1 mutant Arabidopsis, PR genes are constitutively expressed suggesting a negative role of SUMO1 conjugation on PR gene regulation. PR genes are in turn regulated by multiple pathways that may be NPR1 dependent or independent [29]. We checked the expression of PR genes during SyYVCV infection and observed that their levels are the same as in uninfected plants. However, very interestingly, we observed that upon mutating SIM or SUMOylation motifs of βC1, PR gene expression was upregulated 10–15-fold (Additional file 1: Fig. S16B). Regulators of PR genes such as NPR1, TGA and TCP RNAs were also upregulated only in SIM/SUMO motif mutants of βC1 (Additional file 1: Fig. S16A).

In our localization analysis, βC1 is localized in the nucleus, nucleolus and chloroplasts. In our analysis, we observed that most of the viruses producing leaf curl symptoms have arginine instead of lysine at the predicted C-terminal SUMOylation motifs of βC1 (K83 of SyYVCV βC1). Majority of the yellow vein symptoms producing viruses have lysine at the consensus site (Additional file 1: Fig. S17A and17B) (Additional file 2: Table. S2). It is tempting to hypothesize that, in the absence of a chloroplastic transit peptide sequence in βC1, K83 consensus site might be the trigger for chloroplastic localization. Localization data available for βC1 proteins from the Radish leaf curl virus (RaLCV), TYLCCNV and CLCuMuB are in agreement with this hypothesis. RaLCV βC1 was shown to undergo chloroplastic localization along with nuclear localization, whereas TYLCCNV localized in the nucleus and cell membranes and very weakly in the chloroplast. RaLCV has consensus SUMOylation motif similar to that of βC1 (K83, FKQE), whereas TYLCCNB βC1 has a modified motif having arginine instead of lysine (R83, FRQE). CLCuMuB βC1 lacks a consensus SUMOylation motif at the C-terminal end, and it failed to localize in the chloroplast [31, 45]. N-terminal SUMOylation motif mutants (mK18, 24R) did not show any drastic defect in localization, but to our surprise, mutating C-terminal SUMOylation motif (K83 as in mK83R or mK18,24,83R) resulted in a clear defect in chloroplastic localization. Interestingly, nuclear and nucleolar localization was not altered in any of the mutants, indicating the major differences might be in chloroplastic localization of the protein (Fig. 7a).

It is unclear why βC1 localizes to the chloroplast. RNA viruses prefer chloroplast and peroxisomal membranes as the preferential sites for replication. Geminiviruses mostly replicate in the nucleus but their proteins are known to localize in the chloroplast [58]. It had been previously shown that MP from Potato virus Y (*PVY*) and Tobamovirus (*ToMV*) undergo chloroplastic localization by interacting with Rubisco small subunit (*RbSC*) [59]. It was observed in these cases that inhibiting interaction between RbSC and MP or inhibiting chloroplastic localization of MP severely inhibited systemic infection of the virus. From our results, it appears that a similar mechanism might be operating in βC1, as without a chloroplastic transit peptide, βC1 localizes in the chloroplast since triple SUMOylation motif mutant (mK18, 24, 83R) or single C-terminal (mK83R) mutant that failed to localize in the chloroplast also failed to promote systemic viral infection suggesting a direct link between chloroplastic localization of βC1 and viral systemic infection.

Further, it is not clear if chloroplastic localization is a proviral mechanism or antiviral. It is well known that chloroplast has a cascade of specific proteases and chloroplastic proteins undergo degradation in a complex process. The use of protease inhibitors, especially the cysteine protease inhibitor, increased the protein levels of βC1, suggesting that protease-specific degradation of at least a part of the total pool of viral βC1, thus suggesting localization-specific degradation.

Other PTMs might also add another layer of complexity in the regulation of viral proteins. Phosphorylation has been shown to be one such important PTM regulating the effect of other PTMs [17]. Phosphorylation of the serine adjacent to SIM can lead to differential binding of SUMO protein, as has been shown in the analysis of DAXX protein [60]. Interestingly, SIM4 of βC1 has a trailing serine which in our phosphorylation prediction analysis was picked up to be one of the important phosphorylation sites. Further understanding of the multiple PTMs in βC1 might offer insights into the defence and counter-defence in DNA virus-host interactions. Since geminiviruses are closely related to human DNA viruses, the results presented here are likely to offer therapeutic insights to develop novel strategies for control of viral diseases across plants and animals.

## Conclusion

Our results elucidated a novel mechanism by which a geminivirus ensures the steady state level of its pathogenicity determinant protein βC1. We show that the virus is able to modulate its host-mediated degradation targeted through its SIM site by robust SUMOylation. This ensures optimal levels of βC1 during viral infection, so that a pool of active functional protein is always available. βC1 also upregulated global host protein SUMOylation and negatively regulated pathogenicity-related genes, leading to successful viral infection. These results indicate the presence of another layer of defence and counter-defence operating during plant-virus interactions.

## Methods

### Plasmid constructs

The complete genomic sequence of SyYVCV βC1, as well as its amino acid substitution mutants, was cloned from pSD35 harbouring full-length DNA β [33]. The pMAL-p5E (New England Biolabs) and pBIN19 vectors were used as templates to amplify MBP and GFP tags to generate fusion constructs. βC1 and its mutants were cloned in pBIN19 using primers having BamHI and SacI sites generating an N-terminal GFP fusion construct driven by 35S CaMV promoter. As a control, GFP or MBP alone was also cloned into pBIN19 vector which was used a vector control (pBIN). The substitution mutations in βC1 were generated using overlapping PCR primers harbouring the required modification or by site-directed mutagenesis kit (Invitrogen). For recombinant protein expression in *E. coli* and their purification, βC1 and mutants were cloned in pMAL-p5E using NcoI and NotI sites. Vector was modified by adding a precision protease cleavage site in between N-terminal MBP and βC1. For generating infectious clones of DNA β, partial dimers of DNA β having wildtype βC1 ORF or mutants with specific substitutions were designed and synthesized using Geneart (Thermo Fischer) dsDNA service and the fragments were subcloned into pBIN19 binary vector.

Full-length *At*SUMO1, 2, 3 and 5 coding sequences were amplified from cDNA derived from *A. thaliana* young seedlings. *Nb*SUMO1 was amplified from *N. benthamiana* leaves. These amplicons were cloned into expression vector pET-22b (+) (Merck Millipore) carrying N-terminal 6X-HIS tag for protein purification. They were cloned into pBIN19 as fusion proteins either with 3XFLAG or GFP tags translationally fused to the N-terminus of the protein for transient overexpression in plants. Primers used for amplification are listed in Additional file 2: Table S3.

### Protein expression and purification

MBP-tagged SyYVCV βC1 and mutants were transformed in Rosetta Gammi DE3 cells (Novagen) according to the manufacturer’s directions. For purification, 2-l culture at OD 0.7 was induced with 0.1 mM IPTG and incubated at 18 °C for 18 h. Cells were resuspended and lysed in lysis buffer (25 mM Tris-Cl pH 8, 500 mM NaCl, 0.01% tween 20, 5% glycerol, 5 mM 2-Mercaptoethanol, 1 mM PMSF, 1 mg/ml lysozyme and 1x protease inhibitor cocktail [Roche]) using a sonicator with 5 s ‘ON’ and 10 s ‘OFF’ X 10 cycles at 60% amplitude in ice. The clarified supernatant was passed through Dextrin Sepharose column (GE Healthcare), and the unbound protein was removed by washing with a buffer (25 mM Tris-Cl pH 8, 500 mM NaCl, 0.01% tween 20 and 5% glycerol). The protein was eluted using 15 mM maltose. Eluted protein was concentrated and subjected to size exclusion chromatography and buffer exchange to Buffer A (25 mM Tris-Cl pH 8, 150 mM NaCl and 5% glycerol) using HiLoad 16/600200 pg superdex preparative column (GE).

For purification of SUMO proteins, N-terminally His-tagged constructs were transformed in BL21 (DE3) bacterial cells and grown in Luria Bertani (LB) broth. Plant SUMO3 and SUMO5 were insoluble in *E. coli* unlike their mammalian homologs and were purified by an additional denaturation-refolding step. For NMR experiments, uniformly ^13^C/^15^N-labelled *Nb*SUMO was purified using a method described previously [61]. The final protein for backbone assignment was obtained in suspension buffer (50 mM Tris pH 8.0, 100 mM NaCl and 5% glycerol). For NMR experiments, the protein sample was supplemented by 10% D_2_O.

### Transgenic plants and transient expression

Transformation of tobacco (*N. tabacum*, Wisconsin 35) was performed as described previously [62]. Briefly, leaf discs were prepared from 3-week-old *N. tabacum* plants maintained in tissue culture condition, followed by infection with *Agrobacterium* strain LBA4404 (pSB1) harbouring gene of interest [63, 64]. Transformants were selected on kanamycin medium and maintained in greenhouse. For transient overexpression, 3- to 4-week-old *N. tabacum* leaves were infiltrated using *Agrobacterium* LBA4404 (pSB1) strains having appropriate genes suspended in an infiltration buffer (10 mM MES, 10 mM MgCl_2_, pH 5.7 and 100 uM acetosyringone). A culture of 0.6 OD was incubated for 1 h before infiltration. For protein expression studies, an equal amount of culture was infiltrated using a needle-less 1-ml syringe onto 2nd whorl of leaves from the top of either *N. tabacum* or *N. benthamiana*. During sample collection, equal area of infiltrated leaves was collected and further processed for protein expression analysis.

### Plant total protein isolation and western blotting

Total protein was isolated using the acetone-phenol extraction method [65, 66]. Briefly, 200 mg of tissue was ground in liquid nitrogen and protein was precipitated by 10% TCA (Trichloroacetic acid, Sigma) in acetone and the resultant precipitate was pelleted by centrifuging at 13000 rpm for 5 min. Pellet was washed with 0.1 M ammonium acetate in 80% methanol followed by 80% acetone. The final pellet was further extracted using 1:1 ratio SDS extraction buffer and phenol (pH 8.0 Tris-saturated). The resulting mixture was centrifuged at 13000 rpm for 10 min. The protein was precipitated overnight using 0.1 M ammonium acetate in 100% methanol at − 20 °C. Precipitated protein was pelleted (13,000 rpm for 30 min) and was washed once with 100% methanol and then with 80% acetone, air-dried to remove residual acetone and resuspended in 2X SDS lamelli sample loading buffer containing 6 M urea and 1% CHAPS.

For western blot analysis, 20 μg of total protein was loaded either on a 12% Tris-Glycine SDS gel or 4–20% Bio-Rad precast gels. Resolved protein was transferred to nylon membrane (GE (Amersham) Protran, 0.2 μm) and blocked with either 5% blotting grade blocker (Bio-Rad) or 4% BSA (Sigma) in TBS with 0.1% tween 20. Blocked membranes were probed for protein of interest using specific antibody and imaged using Image quant 4000 LAS in chemiluminiscence mode (GE). Blots were stripped using stripping buffer (Restore western stripping buffer, Thermo Fisher) according to the manufacturer’s instructions. Bands were quantified and normalized using FIJI. Antibodies used for WB are listed in Additional file 2: Table S4.

### Immunoprecipitation

Infiltrated leaves expressing the protein of interest were powdered in a mortar under liquid N_2_. About 2 g of tissue was weighed, and to it, 3 volumes of lysis buffer (50 mM Tris-Cl pH 7.4, 150 mM KCL, 1% Triton X100, Protease inhibitor 1 X [Roche], NEM 20uM) were added. The supernatant was collected after a spin at 16000*g* for 30 min and incubated with GFP-Trap (Chromtek) or MBP magnetic beads (NEB) for 3 h at 4 °C. Beads were magnetically separated from the lysate and washed 5 times in wash buffer (50 mM Tris-Cl, pH 7.4; 150 mM KCL, 1 mM PMSF) until the green colour completely disappeared. The final pull-down beads were transferred to a 1.5-ml tube and again washed twice with wash buffer. The 3X SDS sample dye was added to the beads, and the sample was heated at 70 °C for 10 min. The pull-down products were resolved in 4–20% Tris-Glycine SDS gradient gels (Bio-Rad). IP beads used are listed in Additional file 2: Table S4.

### In vitro SUMOylation assay

SUMOylation assay was carried according to a published protocol [61]. For SUMOylation of SyYVCV βC1 protein, 150 nM of substrate protein was incubated with 1 μM E1 (SAE1/SAE2), 2.5 μM E2 (ubc9) and 10 μM of 6x-HIS-tagged *Nb*SUMO1-GG. The reaction was initiated by adding 1 mM ATP. The reaction was carried out at room temperature in a buffer containing 25 mm Tris (pH 8.0), 150 mM NaCl, 5 mM MgCl_2_ and 0.1% Tween 20. The reaction was terminated by adding 2X SDS lamelli buffer, followed by boiling at 95 °C for 1 min and separating on a 4–20% gradient gel. For SUMOylation assay using peptides, 5 μM of peptide substrate was used. For positive control of the reaction and reconfirmation of SUMOylation of βC1, MBP-tagged SyYVCV βC1 and SUMO domain mutants of βC1 were incubated with SUMOylation cascade enzymes as per the manufacturer’s instructions (SUMOylation kit, ENZO life sciences). *Hs*SUMO1 was replaced with *Nb*SUMO1. The reaction was terminated using 2x SDS non-reducing dye and the mixture was resolved in a 4–20% gradient gel (Bio-Rad), followed by detection with anti *At*SUMO1 antibody.

### Inhibitor treatment

Inhibitor treatment was performed as described previously [13]. About 16 h before collection of leaf samples infiltrated with constructs, 50 μM of MG132 (Cellagen) or equal carrier concentration of DMSO (Sigma) were super-infiltrated onto the same leaves. For NEM treatment, 50 μM of NEM in infiltration buffer (MES 10 mM, MgCl_2_ 10 mM) were infiltrated 12 h prior to sample collection. Samples were immediately frozen in liquid N_2_, and proteins were isolated as mentioned above.

### Viral replication assay and southern blotting

Viral titre assay was performed as previously shown [67, 68]. Partial dimer of SyYVCV DNA-A and 35S: SyYVCV βC1 or mutants were mobilized into *Agrobacterium* strain LBA4404 (pSB1) and co-infiltrated in *N. tabacum* leaves. Samples were collected 7 DPI. For checking systemic infection, partial dimer of SyYVCV DNA-A and DNA β or DNA β with mutated βC1 were co-infiltrated in 2-week-old *N. tabacum* or *N. benthamiana* leaves. Genomic DNA from infiltrated and systemic leaves was isolated using the CTAB method [69]. An equal amount of genomic DNA was loaded onto a 0.7% TNE agarose gel and resolved at 5 V/cm. The transfer was performed as previously mentioned [70], and blots were probed with full-length DNA-A or DNA-β, internally labelled with dCTP alpha P32 (BRIT, India) using Rediprime II kit (GE). Blots were scanned using Typhoon Trio Scanner (GE) in phosphorescence mode.

### Yeast two-hybrid screening

All SUMO CDS were translationally fused with the activation domain of pGADT7 AD (Takara Bio). βC1 was fused with binding domain and cloned into pGBKT7 BD (Takara Bio). Plasmids were transformed into AH109 strain as described previously [71] Successful transformants were screened on -Leu, -Trp media followed by screening for interaction on -Leu,- Trp, -His with or without 3AT (Sigma-Aldrich). Successful interactions were further screened on –Leu, -Trp, -His, -Ade media.

### Synthetic peptides

All the synthetic peptides for *Nb*SUMO: SIM titration measurements were purchased from Lifetein LLC as lyophilized powders. The peptides were subsequently dissolved in re-suspension buffer (25 mM Tris pH 8, 100 mM NaCl, 5% glycerol) and used for titration by NMR.

### NMR experiments

All NMR spectra were recorded at 298 K on an 800-MHz Bruker Avance III HD spectrometer equipped with a cryo-probehead. All spectra were processed with NMRpipe [72] and analysed with NMRFAM-SPARKY [73]. For titrations with SIMs, standard 15N-HSQC were recorded for each protein-ligand concentration. Standard triple resonance CBCA (CO) NH, HNCACB, HNCO and HN (CA) CO experiments were used from Bruker library for backbone assignments using a ~ 1 mM uniformly ^13^C,^15^N-labelled *Nb*SUMO. Following peak picking of the backbone experimental data in Sparky, the chemical shift lists were submitted to PINE NMR-server [74], and the assigned peak list was verified and completed manually. For the structure of *Nb*SUMO, the above chemical shift lists were submitted to the CS-Rosetta server along with the primary protein sequence. From the obtained structures, the best PDB was reported out of.

## Supplementary information

**Additional file 1: Fig. S1:** SyYVCV βC1 has multiple conserved SUMOylation sites. **A)** Table summarizing JASSA prediction of SUMOylation sites in βC1. SUMOylation consensus lysine residue is shown in red. PS: represents predictive score. Arrow indicates the direction of the motif. **B)** Heat map showing conserved lysine and predicted SUMOylation sites among βC1 sequences from different viruses. Aligned residue no. indicates the original position of lysine in the protein alignment of βC1. SyYVCV βC1 SUMOylation consensus lysines are highlighted at the top. **Fig. S2**: SyYVCV βC1 can interact with *Nb*SUMO1. **A)** in vitro SUMOylation assay with *Nb*SUMO1-GG and SyYVCV βC1 as substrate using purified SUMOylation cascade enzymes. Concentrations of *Nb*SUMO1 are marked. Red and black triangles represents poly-SUMOylated products and free *Nb*SUMO1, respectively. Black arrow indicates E1-*Nb*SUMO1 conjugate. Right triangle on top of the gel indicates timepoints (30 and 60 min). **B)** Yeast two-hybrid assay with binding domain fused βC1 and activation domain fused *Nb*SUMO1 or *Nb*SUMO1Δ^GG^ (C-terminal di-Glycine deleted, SUMOylation defective), selected in -LW, and screened in -LWH media with 0.2 mM 3AT. Protein marker sizes are indicated in kDa. WB: indicates western blotting using specified antibody. **Fig. S3:** SyYVCV βC1 induces various developmental defects in transgenic plants. **A)** GFP-βC1 expressing transgenic plants showing exerted stigma. Size bar-1 cm. **B)** Histogram representing stigma and pistil measurements. *N* = 10 for each line, two individual lines for GFP-βC1 were analyzed. **C)** Histogram showing height of transgenic GFP-βC1 plants. Numbers on the x-axis indicates transgenic plant line number. *N* = 5/line. **D)** Graph showing early heading date in GFP-βC1 transgenic plants. N = 5/line. Stats: Tukey’s multiple comparison test with *P*-value; four, three and two stars representing *P* ≤ 0.0001, 0.001, 0.01, respectively. **Fig. S4**: SyYVCV βC1 undergoes SUMOylation *in planta*. **A)** Co-IP of GFP-βC1 from transgenic plants using anti-GFP antibody followed by WB. Red arrow represents SUMO1 conjugated βC1. Black arrow shows unmodified βC1. Star shows non-specific band. **B)** Same as in A) except for WB with anti-*At*SUMO1. **C)** WB of transiently over-expressed GFP tagged *Nb*SUMO1 and *Nb*SUMO1^ΔGG^. Red triangle indicates poly-SUMOylated products. Other details are as in Additional file 1: Fig. S2 legend. **Fig. S5:** SyYVCV βC1 weakly interacts with other plant SUMO proteins. **A)** Y2H assay showing interaction of βC1 with plant SUMO proteins. Deletion of di-Glycine motif of SUMO proteins abolishes SUMOylation. Top triangle represents dilutions. **B)** WB analysis of Y2H transformants. AD and BD domain-fused proteins were detected with anti-HA and anti-MYC antibodies, respectively. Black arrow indicates BD fused βC1. Arrow head indicates AD fused plant SUMO proteins. **C)** Co-IP of βC1 with eGFP tagged *Nb*SUMO1, *At*SUMO3 and *At*SUMO5. βC1 and SUMO proteins were co-expressed transiently in *N. tabacum*. βC1 was immuno-precipitated and checked for the presence of conjugated SUMO proteins. About 4–20% denaturing reducing gel was used. The blot was first probed with anti-GFP followed by anti-MBP. Other details are as in Additional file 1: Fig. S2 and S4 legends. **Fig. S6**: SUMOylation of βC1 is required for symptom development. **A)** Top-panel: in-vitro SUMOylation assay using purified SUMO conjugation components and HA tagged βC1 peptides. Lower panel: sequences of peptides. Red and black triangles represent poly-SUMOylated and mono-SUMOylated βC1 peptides, respectively. **B)** Graph showing initiation of flowering in βC1 and SUMO mutant-expressing plants. **C)** Histogram representing height of transgenic GFP- βC1 and βC1 SUMOylation mutant plants. **D)** Graphs showing stamen and pistil measurements in βC1 and its SUMOylation motif mutant plants. Other details are as in Additional file 1: Fig. S2 legend. **Fig. S7:** SUMOylation is required for the stability of βC1 *in planta*. **A)** WB analysis to check stability of transiently over-expressed MBP tagged βC1 and mK18, 24R mutant in *N. tabacum*. Varying concentrations of protein was loaded. Samples were collected at 4 dpi. **B)** Transient protein expression followed by time course analysis of protein levels for MBP-βC1 and its mutant. **C)** Transient expression and WB of individual SUMOylation site lysine mutants of MBP-βC1. Samples were collected at 4 dpi. **D)** Expression of HA tagged βC1 and its SIM, SUMOylation motif mutants in WT yeast (*BY4741*). Black arrow represents MBP-βC1 protein (59 kDa), arrow head represents broken MBP and star represents non-specific band. P: Ponceau staining for total proteins showing RUBISCO. Other details are as in Additional file 1: Fig. S2 legend. **Fig. S8:** SIM sites in SyYVCV βC1 and phylogeny of its potential partner SUMO proteins. **A)** Table showing SIM site prediction in βC1 using JASSA software. Predicted SIM residues are highlighted in blue. P.S. indicates predictive score. **B)** Phylogenetic tree of SUMO proteins. Tree was built in MEGA software using Maximum Likelihood method with 100 bootstraps. **Fig. S9**: Expression analysis and purification of plant SUMO proteins. **A)** Relative expression of *Arabidopsis* SUMO RNAs in various plant tissues of *A. thaliana.* **B)** CBB stained gels showing purified ^15^N labelled recombinant 6X HIS tagged SUMO proteins. **C)** WB with anti-HIS confirming the presence of SUMO5 in pellet. P and S represent pellet and supernatant, respectively. **D-H)** Size exclusion profile of different recombinantly NiNTA purified SUMO proteins on a SD75 column. Void volume 40.25 ml. Additional details are as in Additional file 1: Fig. S2 legend. **Fig. S10**: Sequence of SIM mutants. **A)** Sequence of SIM and mutated SIM peptides used for HSQC experiment. **B)** Table showing predicted SyYVCV βC1 SIM binding sites and various structural and null mutants created in this study. mSIM1, mSIM2,3, mSIM4 and mSIM2,3,4 are null mutants, whereas mS SIM2,3 and mS SIM4 are structural mutants. **Fig. S11**: SIM sites in βC1 C-terminal end interact with *Nb*SUMO1. **A)** The ^15^N-^1^H edited HSQC spectrum of *Nb*SUMO1. The backbone amide assignments are labeled beside the peaks. The glutamine and asparagine side chains are connected by black horizontal lines. **B)** Zoomed HSQC spectrum of *Nb*SUMO1 showing chemical shift in 3 residues (A46, T38 and L43) upon titration with SIM2,3 indicating interaction. Q71, L76 did not show any shift. **C), D), E)** The ^15^N-^1^H HSQC spectrum of *Nb*SUMO1 with different titrations involving βC1 mutated SIMs. C) pmSIM1, D) pmSIM2,3 and E) pmSIM4. No significant interactions were observed during HSQC titrations. **Fig. S12**: SIM sites in SyYVCV βC1 regulate its stability. **A)** Semi-in vivo pulldown assay using purified MBP tagged βC1 and SIM mutants. These were used to pull down GFP tagged *Nb*SUMO1^ΔGG^ from *Nb*SUMO1^ΔGG^ over-expressing plant lysate. **B)** in vitro pull down of 6X-HIS tagged *Nb*SUMO1 using MBP affinity purification by co-incubation with MBP-βC1 or its SIM mutants. **C)** Transient over-expression of MBP tagged βC1, mK18,24R and mK18,24R mSIM2,3 mutant in *N. tabacum* followed by WB with anti-MBP. **D, E)** Graph and representative blot showing transient protein expression followed by time point analysis of protein level of MBP-βC1 and its SUMOylation and SIM mutants. **F)** WB for pull-down product of MBP-βC1, MBP and MBP tagged C-terminal SIM mutant with anti-Ubiquitin detecting poly-ubiquitin. Purple triangle in F) indicates poly-Ub. Additional details are as in Additional file 1: Fig. S2 legend. **Fig. S13**: SIM and SUMOylation motifs of βC1 are necessary for its pathogenicity determinant function. **A)** Southern blot showing viral replication in transgenic plants. Plants were infected using partial dimer of DNA-A on GFP-βC1 or its SUMOylation motif mutant expressing transgenic plants. About 10 μg of genomic DNA was treated with S1 nuclease and was loaded in a TNE gel for Southern blotting. **B)** Same as A) but using SIM mutant plants. Right and left panel represents S1 untreated and treated samples, respectively. **C)** Southern blot showing viral replication assay. Infection was performed with partial dimer of SyYVCV DNA-A along with over-expression of p35S:: GFP-βC1 or individual βC1 SIM motif mutants. About 4 μg of genomic DNA was loaded for blotting. **D)** Systemic infection assay of SyYVCV DNA-A with WT DNA-β and SIM/SUMO mutated βC1 variants incorporated in DNA-β, in local and systemic leaves of *N. benthamiana* at 21 dpi. All samples except D) were collected at 11 dpi for local infection. All blots were probed with full length DNA-A probe. **Fig. S14:** SUMOylation motif in βC1 is important for its subcellular localization. **A)** Localization of GFP tagged βC1. All listed proteins were transiently over-expressed in *N*. *benthamiana* leaf epidermis. Nucleus was stained with DAPI and chlorophyll autofluroscence was measured at 650 nm. βC1 localization in nucleus and nucleolus has been shown with white arrow and a representative nucleus has been zoomed in red box. White box with dashed line represents the zoomed in area from G/C merged panel. (G/D merged) GFP and DAPI filter merged; (G/C) GFP and chlorophyll autofluroscence merged. Vector GFP and βC1 panels were taken for comparison and are same as in Fig. 7. **Fig. S15**: SyYVCV βC1 induces global SUMOylation. **A)** Gel shift assay to check for binding of βC1 or SIM and SUMOylation motif mutants with ssDNA (49 nt). Protein was incubated in increasing concentrations with probe. (+) and (++) represents 2 and 5 μg of protein, respectively. **B)** Global SUMOylation induced by βC1. Total protein was extracted from GFP-βC1 expressing transgenic plants and assessed for *Nb*SUMO1 directed global SUMOylation using anti-*At*SUMO1. **C)** MBP-βC1 or its SIM/SUMO motif mutants were transiently over-expressed in *N. tabacum* and total protein was extracted at 3 dpi. Followed by a WB analysis with anti-*At*SUMO1 antibody to detect *Nb*SUMO1 directed global SUMOylation. **D)** Same as C) except single SIM mutants of MBP-βC1 were used along with double C-terminal SIM mutants. P indicates ponceau staining . **Fig. S16:** SIM and SUMOylation motif of βC1 is essential for host defense suppression. Expression profile of host defense genes in systemic leaves of plants infected with DNA A + β or DNA A + β with βC1 mutated in SIM (mSIM2,3,4) or SUMO motifs (mK18,24,83R). **A)** Expression fold difference of various defense regulator genes. **B)** Expression fold difference of pathogenesis related genes PR1 and PR5. R at the end of sample labels indicates biological replicate. Different letters above each bar indicate significant difference (ANOVA, Tukey-Kramer test, *p* < 0.05). **Fig. S17:** Conservation of SUMOylation sites among viruses producing similar symptoms. **A)** Seqlogo of begomoviral βC1 sequences mostly associated with leaf curl symptoms. **B)** βC1 sequences derived from viruses associated with leaf yellowing/mosaic symptoms. **Fig. S18:** Uncropped images of blots in main Fig. 1, 2, 3, 4, 5, 6, 7 and replicates of western blots. **Fig. S19:** Uncropped images of blots in Fig. S1–16.

**Additional file 2: Table S1:** Accession numbers used for building alignments. **Table S2:** Accession numbers used for K83 site analysis. **Table S3:** List of primers used in this study. **Table S4:** List of antibodies and materials used for IP.

## Data Availability

All data generated or analysed during this study are included in this published article (and its supplementary information files)**.** Assigned chemical shift list of *Nb*SUMO1 has been submitted to BMRB (Biological Magnetic Resonance Bank) [75]. BMRB entry accession number: ‘50142’. (http://www.bmrb.wisc.edu/data_library/summary/index.php?bmrbId=50142).

## References

[1] Hanley-Bowdoin L, Bejarano ER, Robertson D, Mansoor S (2013). Geminiviruses: masters at redirecting and reprogramming plant processes. Nat Rev Microbiol.

[2] Heyraud-nitschke F, Schumacher S, Laufs J, Schaefer S, Schell J, Gronenborn B (1995). Determination of the origin cleavage and joining domain of geminivirus rep proteins. Nucleic Acids Res.

[3] Muñoz-Martín A, Collin S, Herreros E, Mullineaux PM, Fernández-Lobato M, Fenoll C (2003). Regulation of MSV and WDV virion-sense promoters by WDV nonstructural proteins: a role for their retinoblastoma protein-binding motifs. Virology.

[4] Trinks D, Rajeswaran R, Shivaprasad PV, Akbergenov R, Oakeley EJ, Veluthambi K (2005). Suppression of RNA silencing by a geminivirus nuclear protein, AC2, correlates with transactivation of host genes. J Virol.

[5] Stanley J, Latham JR (1992). A symptom variant of beet curly top geminivirus produced by mutation of open reading frame C4. Virology.

[6] Stanley J, Latham JR, Pinner MS, Bedford I, Markham PG (1992). Mutational analysis of the monopartite geminivirus beet curly top virus. Virology.

[7] Ribet D, Cossart P (2010). Post-translational modifications in host cells during bacterial infection. FEBS Lett.

[8] Jakubiec A, Tournier V, Drugeon G, Pflieger S, Camborde L, Vinh J (2006). Phosphorylation of viral RNA-dependent RNA polymerase and its role in replication of a plus-strand RNA virus. J Biol Chem.

[9] Shen Q, Liu Z, Song F, Xie Q, Hanley-Bowdoin L, Zhou X (2011). Tomato S1SnRK1 protein interacts with and phosphorylates βC1, a pathogenesis protein encoded by a geminivirus β-satellite. Plant Physiol.

[10] Deom CM, Lapidot M, Beachy RN (1992). Plant virus movement proteins. Cell.

[11] Reichel C, Beachy RN (2000). Degradation of tobacco mosaic virus movement protein by the 26S proteasome. J Virol.

[12] Jia Q, Liu N, Xie K, Dai Y, Han S, Zhao X (2016). CLCuMuB βC1 subverts ubiquitination by interacting with NbSKP1s to enhance geminivirus infection in Nicotiana benthamiana. PLoS Pathog.

[13] Shen Q, Hu T, Bao M, Cao L, Zhang H, Song F (2016). Tobacco RING E3 ligase NtRFP1 mediates ubiquitination and proteasomal degradation of a geminivirus-encoded βC1. Mol Plant.

[14] Haxim Y, Ismayil A, Jia Q, Wang Y, Zheng X, Chen T, et al. Autophagy functions as an antiviral mechanism against geminiviruses in plants. Elife. 2017;6. 10.7554/eLife.23897. 10.7554/eLife.23897 PMC5362266 28244873

[15] Mei Y, Wang Y, Hu T, Yang X, Lozano-Duran R, Sunter G (2018). Nucleocytoplasmic shuttling of geminivirus C4 protein mediated by phosphorylation and myristoylation is critical for viral pathogenicity. Mol Plant.

[16] Florentino LH, Santos AA, Fontenelle MR, Pinheiro GL, Zerbini FM, Baracat-Pereira MC (2006). A PERK-like receptor kinase interacts with the geminivirus nuclear shuttle protein and potentiates viral infection. J Virol.

[17] Saleh A, Withers J, Mohan R, Marqués J, Gu Y, Yan S (2015). Posttranslational modifications of the master transcriptional regulator NPR1 enable dynamic but tight control of plant immune responses. Cell Host Microbe.

[18] Chau V, Tobias J, Bachmair A, Marriott D, Ecker D, Gonda D (1989). A multiubiquitin chain is confined to specific lysine in a targeted short-lived protein. Science (80- ).

[19] Deng L, Wang C, Spencer E, Yang L, Braun A, You J (2000). Activation of the IκB kinase complex by TRAF6 requires a dimeric ubiquitin-conjugating enzyme complex and a unique polyubiquitin chain. Cell.

[20] Johnson LN, Barford D (1993). The effects of phosphorylation on the structure and function of proteins. Annu Rev Biophys Biomol Struct.

[21] Park HJ, Kim W-Y, Park HC, Lee SY, Bohnert HJ, Yun D-J (2011). SUMO and SUMOylation in plants. Mol Cells.

[22] Nayak A, Müller S (2014). SUMO-specific proteases/isopeptidases: SENPs and beyond. Genome Biol.

[23] Lois LM (2010). Diversity of the SUMOylation machinery in plants. Biochem Soc Trans.

[24] Bernier-Villamor V, Sampson DA, Matunis MJ, Lima CD (2002). Structural basis for E2-mediated SUMO conjugation revealed by a complex between ubiquitin-conjugating enzyme Ubc9 and RanGAP1. Cell.

[25] Song J, Durrin LK, Wilkinson TA, Krontiris TG, Chen Y (2004). Identification of a SUMO-binding motif that recognizes SUMO-modified proteins. Proc Natl Acad Sci.

[26] Beauclair G, Bridier-Nahmias A, Zagury J-F, Saïb A, Zamborlini A (2015). JASSA: a comprehensive tool for prediction of SUMOylation sites and SIMs. Bioinformatics.

[27] Kerscher O (2007). SUMO junction—what’s your function?. EMBO Rep.

[28] Parker JL, Bucceri A, Davies AA, Heidrich K, Windecker H, Ulrich HD (2008). SUMO modification of PCNA is controlled by DNA. EMBO J.

[29] Lee J, Nam J, Park HC, Na G, Miura K, Jin JB, et al. Salicylic acid-mediated innate immunity in Arabidopsis is regulated by SIZ1 SUMO E3 ligase. Plant J. 2006;49:79–90. 10.1111/j.1365-313X.2006.02947.x. 10.1111/j.1365-313X.2006.02947.x 17163880

[30] Cheng X, Xiong R, Li Y, Li F, Zhou X, Wang A (2017). Sumoylation of turnip mosaic virus RNA polymerase promotes viral infection by counteracting the host NPR1-mediated immune response. Plant Cell.

[31] Cui X, Li G, Wang D, Hu D, Zhou X (2005). A Begomovirus DNA-encoded protein binds DNA, functions as a suppressor of RNA silencing, and targets the cell nucleus. J Virol.

[32] Covey SN, Al-Kaff NS, Lángara A, Turner DS (1997). Plants combat infection by gene silencing. Nature.

[33] Das S, Hegde A, Shivaprasad PV (2018). Molecular characterization of a new begomovirus infecting Synedrella nodiflora in South India. Arch Virol.

[34] Zhao Q, Xie Y, Zheng Y, Jiang S, Liu W, Mu W (2014). GPS-SUMO: a tool for the prediction of sumoylation sites and SUMO-interaction motifs. Nucleic Acids Res.

[35] Matic I, Schimmel J, Hendriks IA, van Santen MA, van de Rijke F, van Dam H (2010). Site-specific identification of SUMO-2 targets in cells reveals an inverted SUMOylation motif and a hydrophobic cluster SUMOylation motif. Mol Cell.

[36] Amin I, Patil BL, Briddon RW, Mansoor S, Fauquet CM (2011). Comparison of phenotypes produced in response to transient expression of genes encoded by four distinct begomoviruses in Nicotiana benthamiana and their correlation with the levels of developmental miRNAs. Virol J.

[37] Bazzini AA, Hopp HE, Beachy RN, Asurmendi S (2007). Infection and coaccumulation of tobacco mosaic virus proteins alter microRNA levels, correlating with symptom and plant development. Proc Natl Acad Sci.

[38] Colby T, Matthäi A, Boeckelmann A, Stuible H-P (2006). SUMO-conjugating and SUMO-deconjugating enzymes from Arabidopsis. Plant Physiol.

[39] Kurepa J, Walker JM, Smalle J, Gosink MM, Davis SJ, Durham TL (2003). The small ubiquitin-like modifier (SUMO) protein modification system in Arabidopsis. J Biol Chem.

[40] Brückner A, Polge C, Lentze N, Auerbach D, Schlattner U (2009). Yeast two-hybrid, a powerful tool for systems biology. Int J Mol Sci.

[41] Dahlmann B, Kuehn L, Rutschmann M, Reinauer H (1985). Purification and characterization of a multicatalytic high-molecular-mass proteinase from rat skeletal muscle. Biochem J.

[42] Lange OF, Rossi P, Sgourakis NG, Song Y, Lee H-W, Aramini JM (2012). Determination of solution structures of proteins up to 40 kDa using CS-Rosetta with sparse NMR data from deuterated samples. Proc Natl Acad Sci.

[43] Tripathi V, Chatterjee KS, Das R (2019). Casein kinase-2–mediated phosphorylation increases the SUMO-dependent activity of the cytomegalovirus transactivator IE2. J Biol Chem.

[44] Eini O, Dogra S, Selth LA, Dry IB, Randles JW, Rezaian MA. Interaction with a host ubiquitin-conjugating enzyme is required for the pathogenicity of a geminiviral DNA β satellite. Mol Plant-Microbe Interact. 2009;22:737–46. 10.1094/MPMI-22-6-0737 19445598

[45] Bhattacharyya D, Gnanasekaran P, Kumar RK, Kushwaha NK, Sharma VK, Yusuf MA (2015). A geminivirus betasatellite damages the structural and functional integrity of chloroplasts leading to symptom formation and inhibition of photosynthesis. J Exp Bot.

[46] Shen W, Dallas MB, Goshe MB, Hanley-Bowdoin L (2014). SnRK1 phosphorylation of AL2 delays cabbage leaf curl virus infection in Arabidopsis. J Virol.

[47] Li H, Zeng R, Chen Z, Liu X, Cao Z, Xie Q (2018). S-acylation of a geminivirus C4 protein is essential for regulating the CLAVATA pathway in symptom determination. J Exp Bot.

[48] Kushwaha NK, Bhardwaj M, Chakraborty S (2017). The replication initiator protein of a geminivirus interacts with host monoubiquitination machinery and stimulates transcription of the viral genome. PLoS Pathog.

[49] Chowda-Reddy RV, Achenjang F, Felton C, Etarock MT, Anangfac M-T, Nugent P (2008). Role of a geminivirus AV2 protein putative protein kinase C motif on subcellular localization and pathogenicity. Virus Res.

[50] Tsutakawa SE, Yan C, Xu X, Weinacht CP, Freudenthal BD, Yang K (2015). Structurally distinct ubiquitin- and Sumo-modified PCNA: implications for their distinct roles in the DNA damage response. Structure.

[51] Sianipar IR, Matsui C, Minami N, Gan X, Deng L, Hotta H (2015). Physical and functional interaction between hepatitis C virus NS5A protein and ovarian tumor protein deubiquitinase 7B. Microbiol Immunol.

[52] Domingues P, Golebiowski F, Tatham MH, Lopes AM, Taggart A, Hay RT (2015). Global reprogramming of host SUMOylation during influenza virus infection. Cell Rep.

[53] Boggio R, Colombo R, Hay RT, Draetta GF, Chiocca S (2004). A mechanism for inhibiting the SUMO pathway. Mol Cell.

[54] De La Cruz-Herrera CF, Shire K, Siddiqi UZ, Frappier L (2018). A genome-wide screen of Epstein-Barr virus proteins that modulate host SUMOylation identifies a SUMO E3 ligase conserved in herpesviruses. PLoS Pathog.

[55] Sanchez-Duran MA, Dallas MB, Ascencio-Ibanez JT, Reyes MI, Arroyo-Mateos M, Ruiz-Albert J (2011). Interaction between geminivirus replication protein and the SUMO-conjugating enzyme is required for viral infection. J Virol.

[56] Rytz TC, Miller MJ, McLoughlin F, Augustine RC, Marshall RS, Juan Y, et al. SUMOylome Profiling Reveals a Diverse Array of Nuclear TargetsModified by the SUMO Ligase SIZ1 during Heat Stress. Plant Cell. 2018;30:1077–99. 10.1105/tpc.17.00993. 10.1105/tpc.17.00993 PMC6002191 29588388

[57] Murtas G, Reeves PH, Fu YF, Bancroft I, Dean C, Coupland G. A Nuclear Protease Required for Flowering-Time Regulation in Arabidopsis Reduces the Abundance of SMALL UBIQUITIN-RELATED MODIFIER Conjugates. Plant Cell. 2003;15:2308–19. 10.1105/tpc.015487 PMC197297 14507998

[58] Gutierrez C (1999). Geminivirus DNA replication. Cell Mol Life Sci.

[59] Zhao J, Liu Q, Zhang H, Jia Q, Hong Y, Liu Y (2013). The Rubisco small subunit is involved in Tobamovirus movement and Tm-2 2-mediated extreme resistance. Plant Physiol.

[60] Chang C-C, Naik MT, Huang Y-S, Jeng J-C, Liao P-H, Kuo H-Y (2011). Structural and functional roles of Daxx SIM phosphorylation in SUMO Paralog-selective binding and apoptosis modulation. Mol Cell.

[61] Chatterjee KS, Tripathi V, Das R (2019). A conserved and buried edge-to-face aromatic interaction in small ubiquitin-like modifier (SUMO) has a role in SUMO stability and function. J Biol Chem.

[62] Sunilkumar G, Vijayachandra K, Veluthambi K (1999). Preincubation of cut tobacco leaf explants promotes Agrobacterium-mediated transformation by increasing vir gene induction. Plant Sci.

[63] Stachel SE, Zambryski PC (1986). Agrobacterium tumefaciens and the susceptible plant cell: a novel adaptation of extracellular recognition and DNA conjugation. Cell.

[64] Yanofsky MF, Porter SG, Young C, Albright LM, Gordon MP, Nester EW (1986). The virD operon of Agrobacterium tumefaciens encodes a site-specific endonuclease. Cell.

[65] Wang W, Vignani R, Scali M, Cresti M (2006). A universal and rapid protocol for protein extraction from recalcitrant plant tissues for proteomic analysis. Electrophoresis.

[66] Tirumalai V, Swetha C, Nair A, Pandit A, Shivaprasad PV (2019). miR828 and miR858 regulate VvMYB114 to promote anthocyanin and flavonol accumulation in grapes. J Exp Bot.

[67] Shivaprasad PV, Rajeswaran R, Blevins T, Schoelz J, Meins F, Hohn T (2008). The CaMV transactivator/viroplasmin interferes with RDR6-dependent trans-acting and secondary siRNA pathways in Arabidopsis. Nucleic Acids Res.

[68] Shivaprasad PV, Thomas M, Balamani V, Biswas D, Vanitharani R, Karthikeyan AS (2006). Factors contributing to deletion within Mungbean yellow mosaic virus partial dimers in binary vectors used for agroinoculation. J Virol Methods.

[69] Rogers SO, Bendich AJ (1994). Extraction of total cellular DNA from plants, algae and fungi. Plant molecular biology manual.

[70] Shivaprasad PV, Thillaichidambaram P, Balaji V, Veluthambi K (2006). Expression of full-length and truncated Rep genes from Mungbean yellow mosaic virus-Vigna inhibits viral replication in transgenic tobacco. Virus Genes.

[71] Gietz RD, Woods RA (2002). Transformation of yeast by lithium acetate/single-stranded carrier DNA/polyethylene glycol method. Methods Enzymol.

[72] Delaglio F, Grzesiek S, Vuister G, Zhu G, Pfeifer J, Bax A. NMRPipe: a multidimensional spectral processing system based on UNIX pipes. J Biomol NMR. 1995;6. 10.1007/BF00197809. 10.1007/BF00197809 8520220

[73] Lee W, Tonelli M, Markley JL (2015). NMRFAM-SPARKY: enhanced software for biomolecular NMR spectroscopy. Bioinformatics.

[74] Bahrami A, Assadi AH, Markley JL, Eghbalnia HR (2009). Probabilistic interaction network of evidence algorithm and its application to complete labeling of peak lists from protein NMR spectroscopy. PLoS Comput Biol.

[75] Ulrich EL, Akutsu H, Doreleijers JF, Harano Y, Ioannidis YE, Lin J, et al. BioMagResBank. Nucleic Acids Res. 2007;36Database:D402–8.10.1093/nar/gkm957. 10.1093/nar/gkm957 PMC2238925 17984079

