# Supplementary material for: Stability of Begomoviral pathogenicity determinant βC1 is modulated by mutually antagonistic SUMOylation and SIM interactions
Source: BMC Biol. 2020 Aug 31;18:110. doi: 10.1186/s12915-020-00843-y (PMC7461331; doi:10.1186/s12915-020-00843-y)
Supplement: Supplementary file 1 — Additional file 1: Fig. S1: SyYVCV βC1 has multiple conserved SUMOylation sites. A) Table summarizing JASSA prediction of SUMOylation sites in βC1. SUMOylation consensus lysine residue is shown in red. PS: represents predictive score. Arrow indicates the direction of the motif. B) Heat map showing conserved lysine and predicted SUMOylation sites among βC1 sequences from different viruses. Aligned residue no. indicates the original position of lysine in the protein alignment of βC1. SyYVCV βC1 SUMOylation consensus lysines are highlighted at the top. Fig. S2: SyYVCV βC1 can interact with NbSUMO1. A) in vitro SUMOylation assay with NbSUMO1-GG and SyYVCV βC1 as substrate using purified SUMOylation cascade enzymes. Concentrations of NbSUMO1 are marked. Red and black triangles represents poly-SUMOylated products and free NbSUMO1, respectively. Black arrow indicates E1-NbSUMO1 conjugate. Right triangle on top of the gel indicates timepoints (30 and 60 min). B) Yeast two-hybrid assay with binding domain fused βC1 and activation domain fused NbSUMO1 or NbSUMO1ΔGG (C-terminal di-Glycine deleted, SUMOylation defective), selected in -LW, and screened in -LWH media with 0.2 mM 3AT. Protein marker sizes are indicated in kDa. WB: indicates western blotting using specified antibody. Fig. S3: SyYVCV βC1 induces various developmental defects in transgenic plants. A) GFP-βC1 expressing transgenic plants showing exerted stigma. Size bar-1 cm. B) Histogram representing stigma and pistil measurements. N = 10 for each line, two individual lines for GFP-βC1 were analyzed. C) Histogram showing height of transgenic GFP-βC1 plants. Numbers on the x-axis indicates transgenic plant line number. N = 5/line. D) Graph showing early heading date in GFP-βC1 transgenic plants. N = 5/line. Stats: Tukey’s multiple comparison test with P-value; four, three and two stars representing P ≤ 0.0001, 0.001, 0.01, respectively. Fig. S4: SyYVCV βC1 undergoes SUMOylation in planta. A) Co-IP of GFP-βC1 from tran [file 12915_2020_843_MOESM1_ESM.pdf]

Stability of Begomoviral pathogenicity determinant βC1 is modulated by mutually antagonistic SUMOylation and SIM interactions

Ashwin Nair[1, 2], Kiran Sankar Chatterjee[1], Vikram Jha[1, 3], Ranabir Das[1], and P. V. Shivaprasad[1*]

This file contains following information:

Additional File 1: Fig.S1-19

Fig.S1: SyYVCV βC1 has multiple conserved SUMOylation sites.

Fig.S2: SyYVCV βC1 can interact with *Nb*SUMO1.

Fig.S3: SyYVCV βC1 induces various developmental defects in transgenic plants.

Fig.S4: SyYVCV βC1 undergoes SUMOylation *in planta*.

Fig.S5: SyYVCV βC1 weakly interacts with other plant SUMO proteins.

Fig.S6: SUMOylation of βC1 is required for symptom development.

Fig.S7: SUMOylation is required for the stability of βC1 *in planta*.

Fig.S8: SIM sites in SyYVCV βC1 and phylogeny of its potential partner SUMO proteins.

Fig.S9: Expression analysis and purification of plant SUMO proteins.

Fig.S10: Sequence of SIM mutants.

Fig.S11: SIM sites in βC1 C-terminal end interact with *Nb*SUMO1.

Fig.S12: SIM sites in SyYVCV βC1 regulate its stability.

Fig.S13: SIM and SUMOylation motifs of βC1 are necessary for its pathogenicity determinant function.

Fig.S14: SUMOylation motif in βC1 is important for its subcellular localization.

Fig.S15: SyYVCV βC1 induces global SUMOylation.

Fig.S16: SIM and SUMOylation motif of βC1 is essential for host defense suppression

Fig.S17: Conservation of SUMOylation sites among viruses producing similar symptoms.

Fig.S18: Uncropped images of blots in main figures 1-7 and replicates of western blots.

Fig.S19: Uncropped images of blots in Supplementary Figures 1-16.

A)

Site	AA	Sequence	Type	P.S.
Ss1	**K18**	F I V D V **K** L M Q E D	INV. consensus	Low
Ss2	**K24**	K L M Q E D **K** I S V Q I	INV. consensus	High
Ss3	**K83**	T I G E F **K** Q E D M I E	Consensus	Low

B)

Conserved SUMOylation sites

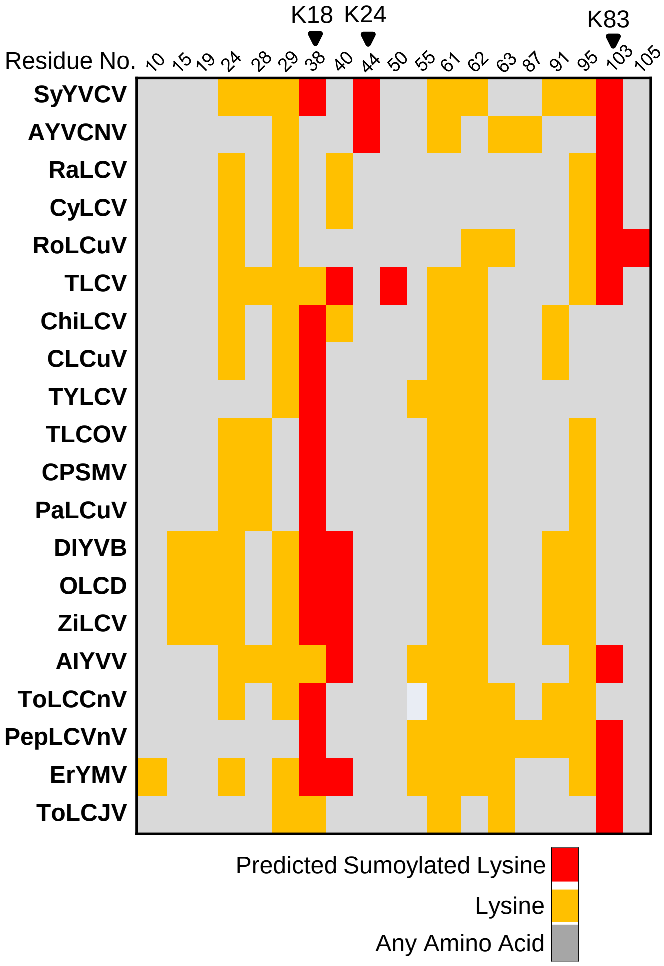

Fig.S1: SyYVCV βC1 has multiple conserved SUMOylation sites.

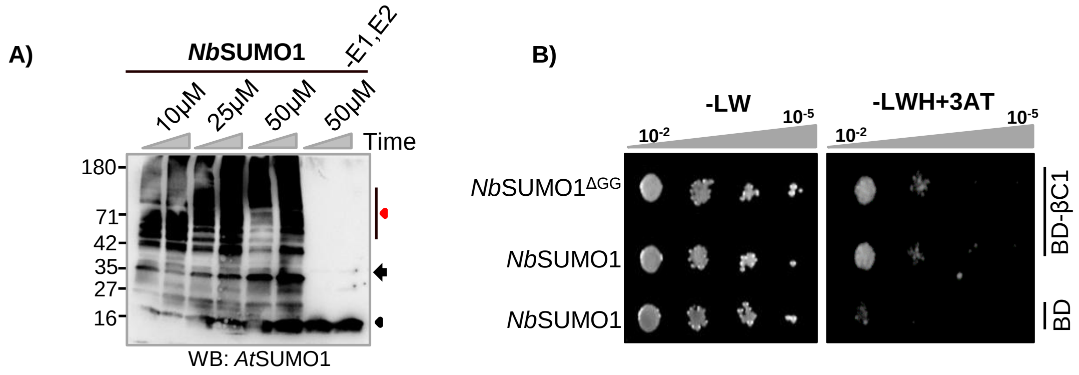

Fig.S2: SyYVCV βC1 can interact with *Nb*SUMO1.

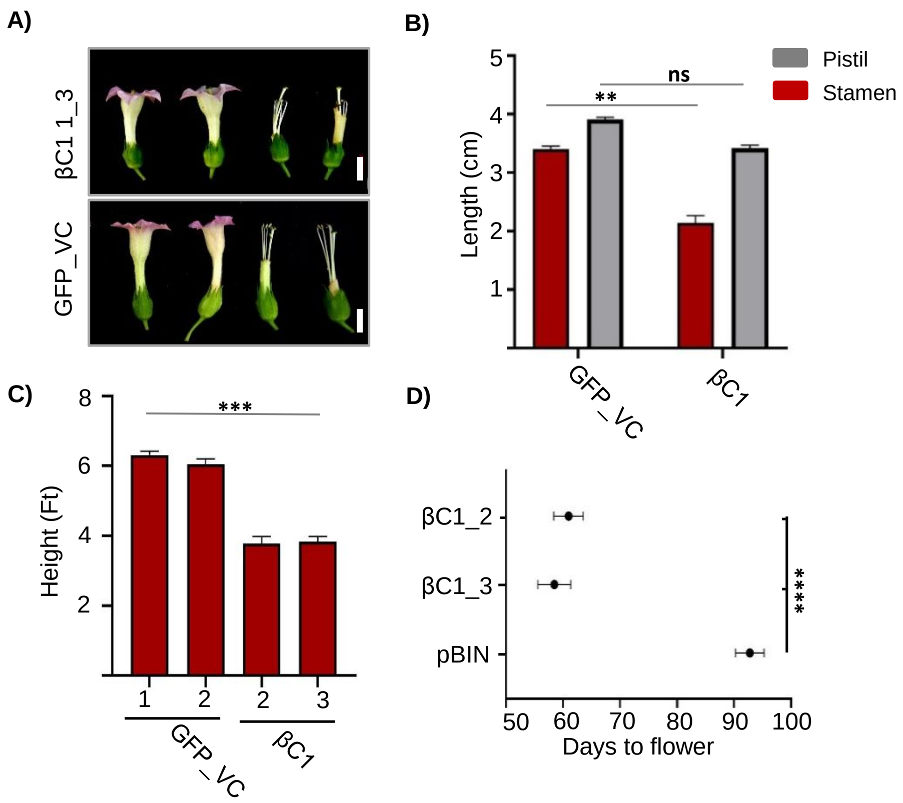

Fig.S3: SyYVCV βC1 induces various developmental defects in transgenic plants.

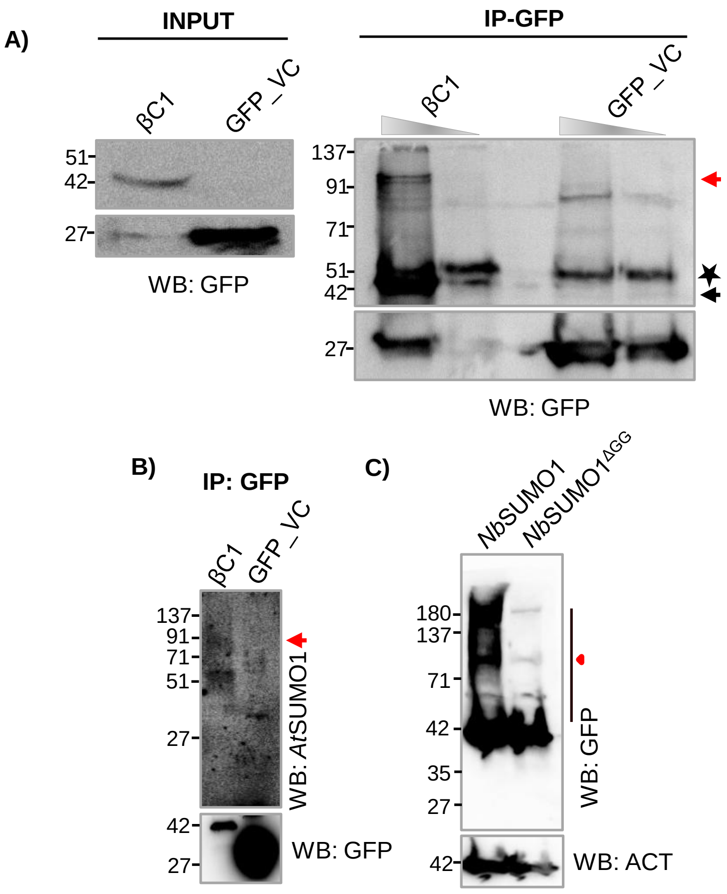

Fig.S4: SyYVCV βC1 undergoes SUMOylation *in planta*.

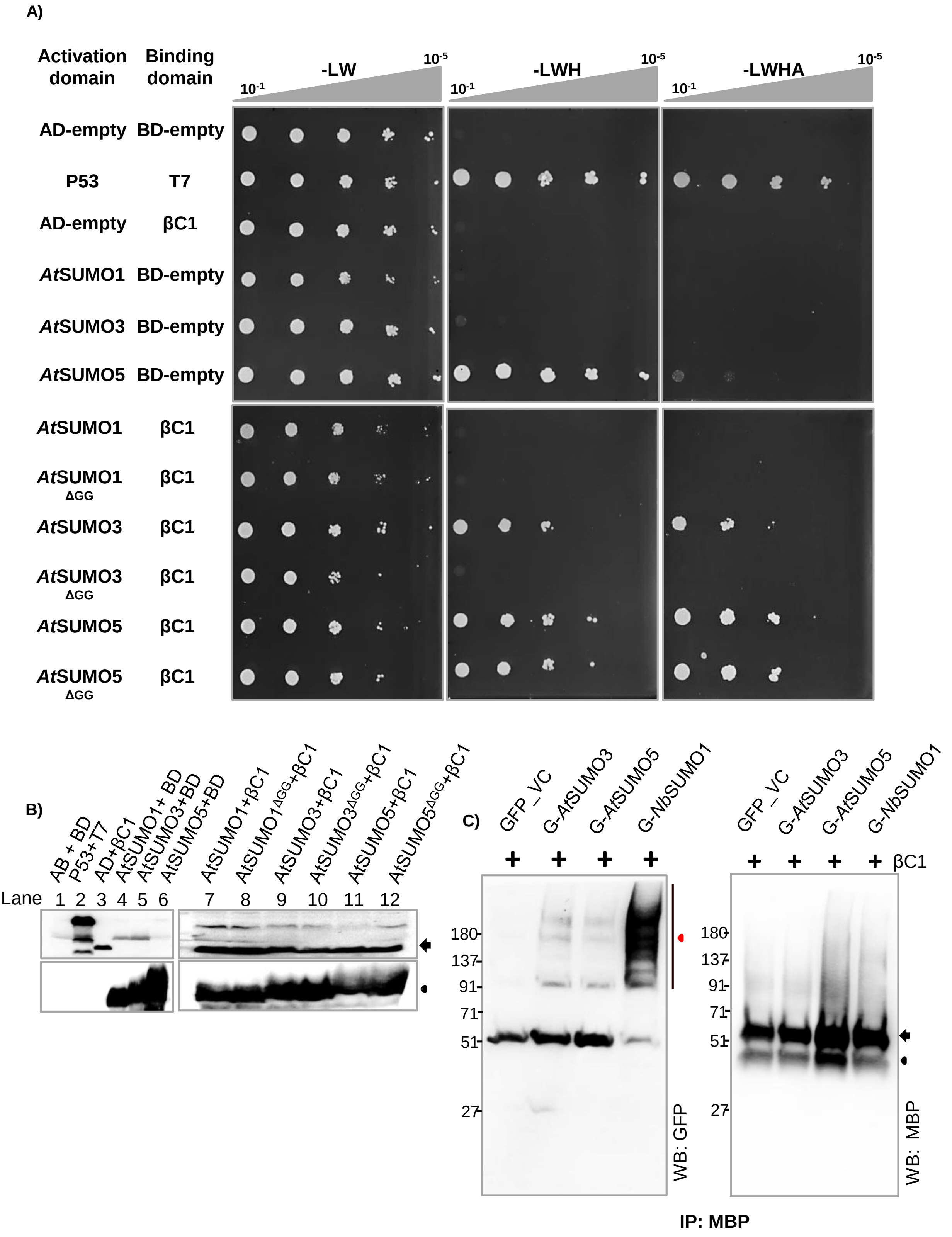

Fig.S5: SyYVCV βC1 weakly interacts with other plant SUMO proteins.

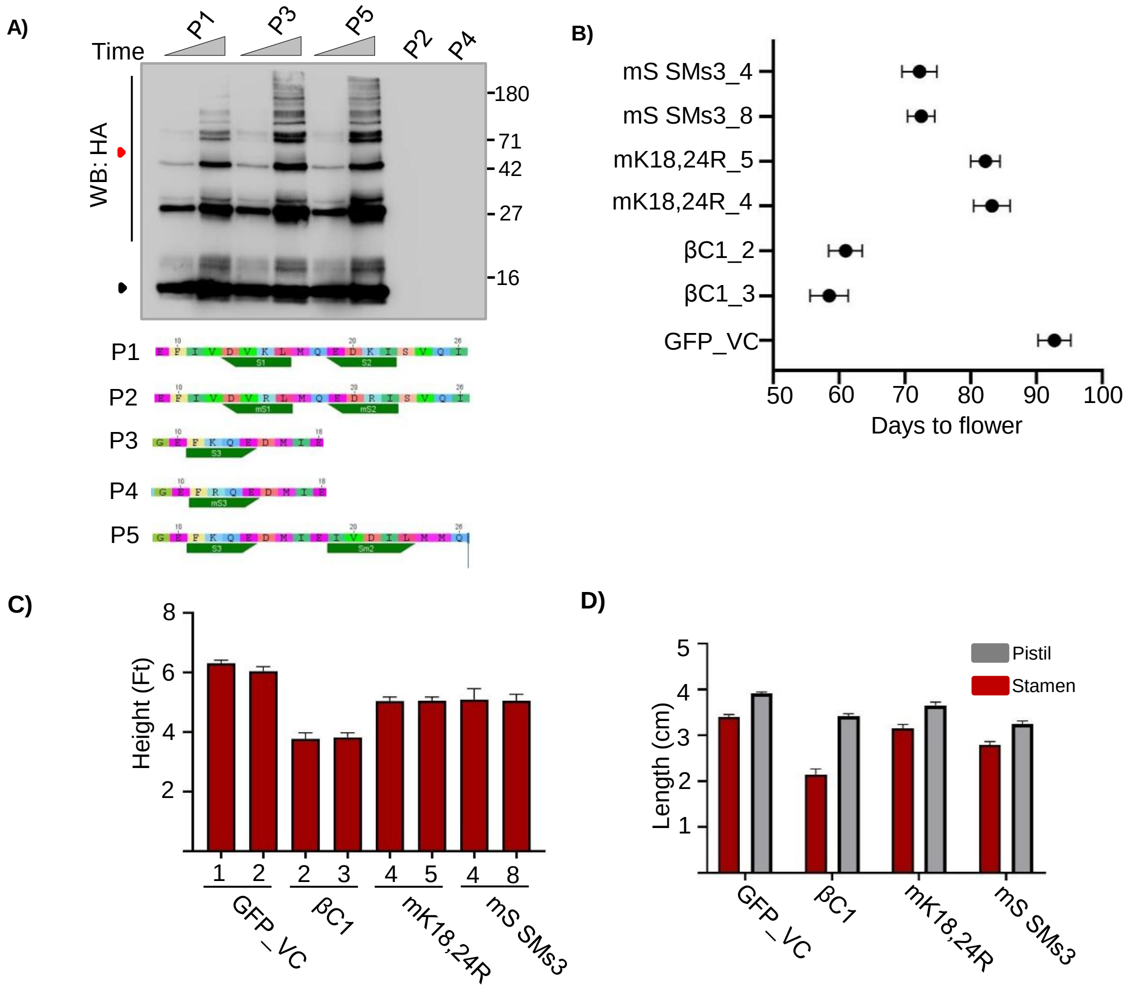

Fig.S6: SUMOylation of βC1 is required for symptom development.

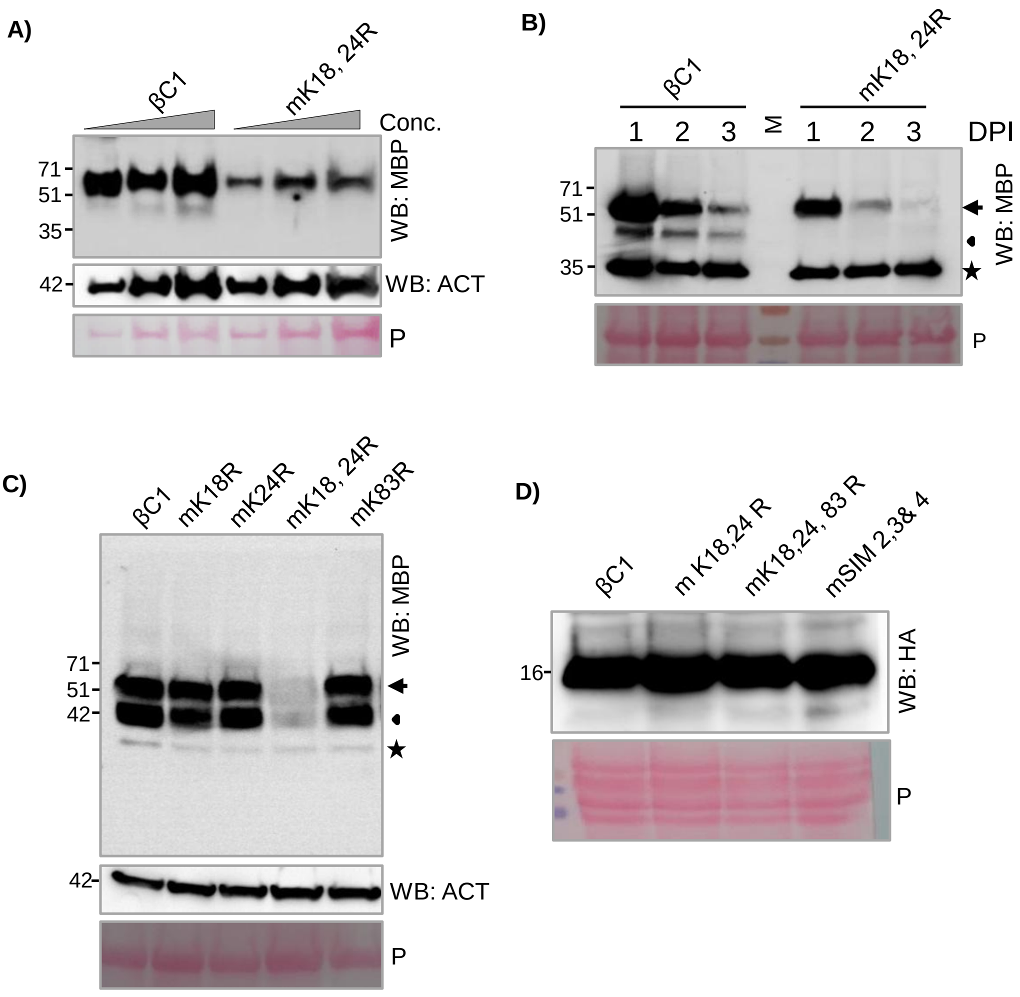

Fig.S7: SUMOylation is required for the stability of βC1 *in planta*.

A)

Domain	Sequence	Type	PS	AA No.
SIM s1	G M E F I V D V K L M Q	SIM β	4.191	**14-17**
SIM s2	D M I E I V D I L M M Q E	SIM β	4.501	**90-93**
SIM s3	M I E I V D I L M M Q E	SIM 2	0.918	**91-94**
SIM s4	Q E A P V I D I N V S D E	SIM β	24.07	**101-104**

B)

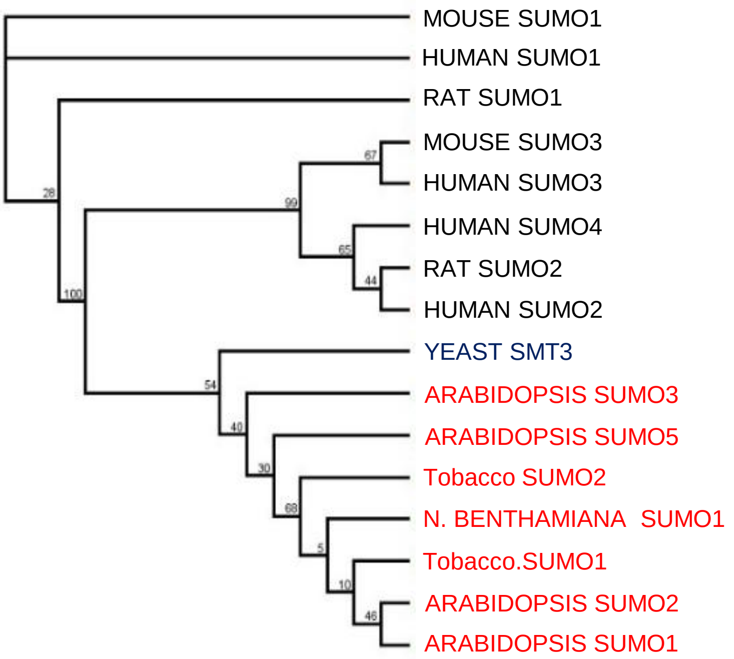

Fig.S8: SIM sites in SyYVCV βC1 and phylogeny of its potential partner SUMO proteins.

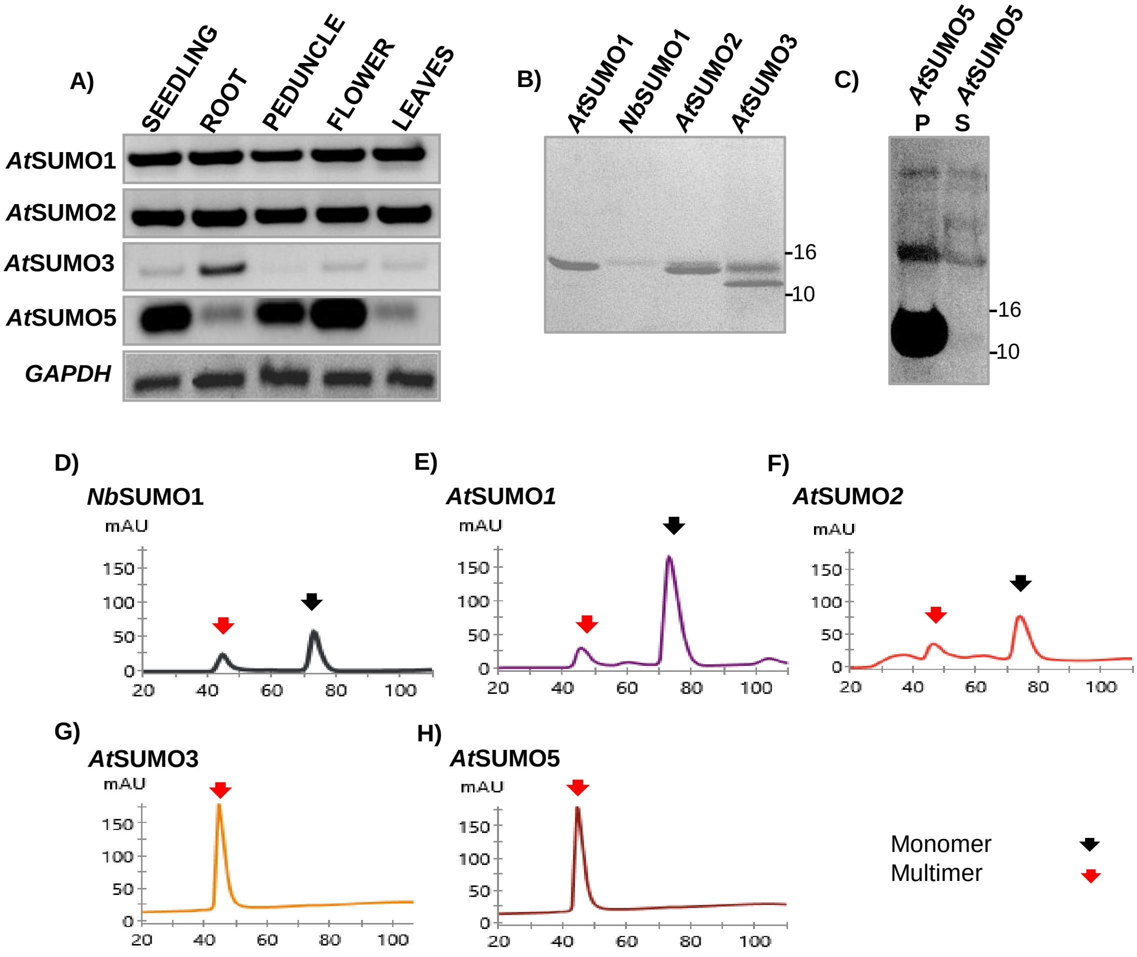

Fig.S9: Expression analysis and purification of plant SUMO proteins.

A)

pSIM1	: KKGMEFIVDVKLMQEDKI
pmSIM1	: WKKGMEFAADAKLMQEDKI
pSIM 2,3	: KQEDMIEIVDILMMQEAPVI
pmSIM2,3	: WKQEDMIEIADAAMMQEAPVI
pSIM4	: ILMMQEAPVIDINVSDEYEV
pmSIM4	: WILMMQEAPAADANVSDEYEV

B)

Residue No.	14 18	90 95	101 105	118
SyYVCV βC1	M - F I VDV K -	I E I VDI L -	P VI DI N -	V
mSIM 1	M - F AAAA K -	I E I VDI L -	P VI DI N -	V
mSIM 2,3	M - F I VDV K -	I E AAAA L -	P VI DI N -	V
mSIM 4	M - F I VDV K -	I E I VDI L -	P AAAA N -	V
mSIM 2,3,4	M - F I VDV K -	I E AAAA L -	P AAAA N -	V
mS SIM 2,3	M - F I VDV K -	I E I KAI L -	P VI DI N -	V
mS SIM 4	M - F I VDV K -	I E I VDI L -	P VI SG N -	V

Fig.S10: Sequence of SIM mutants.

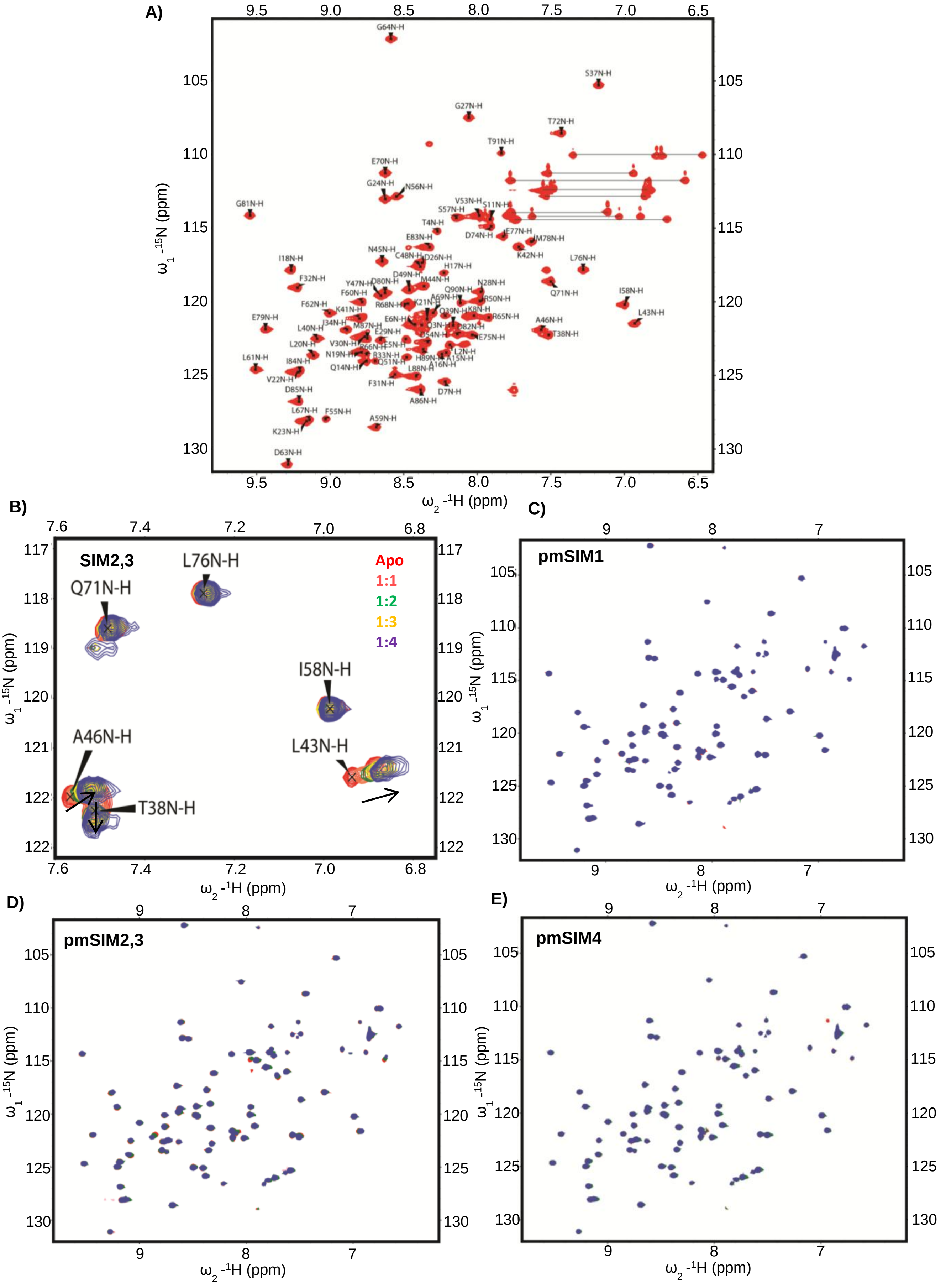

Fig.S11: SIM sites in βC1 C-terminal end interact with *Nb*SUMO1.

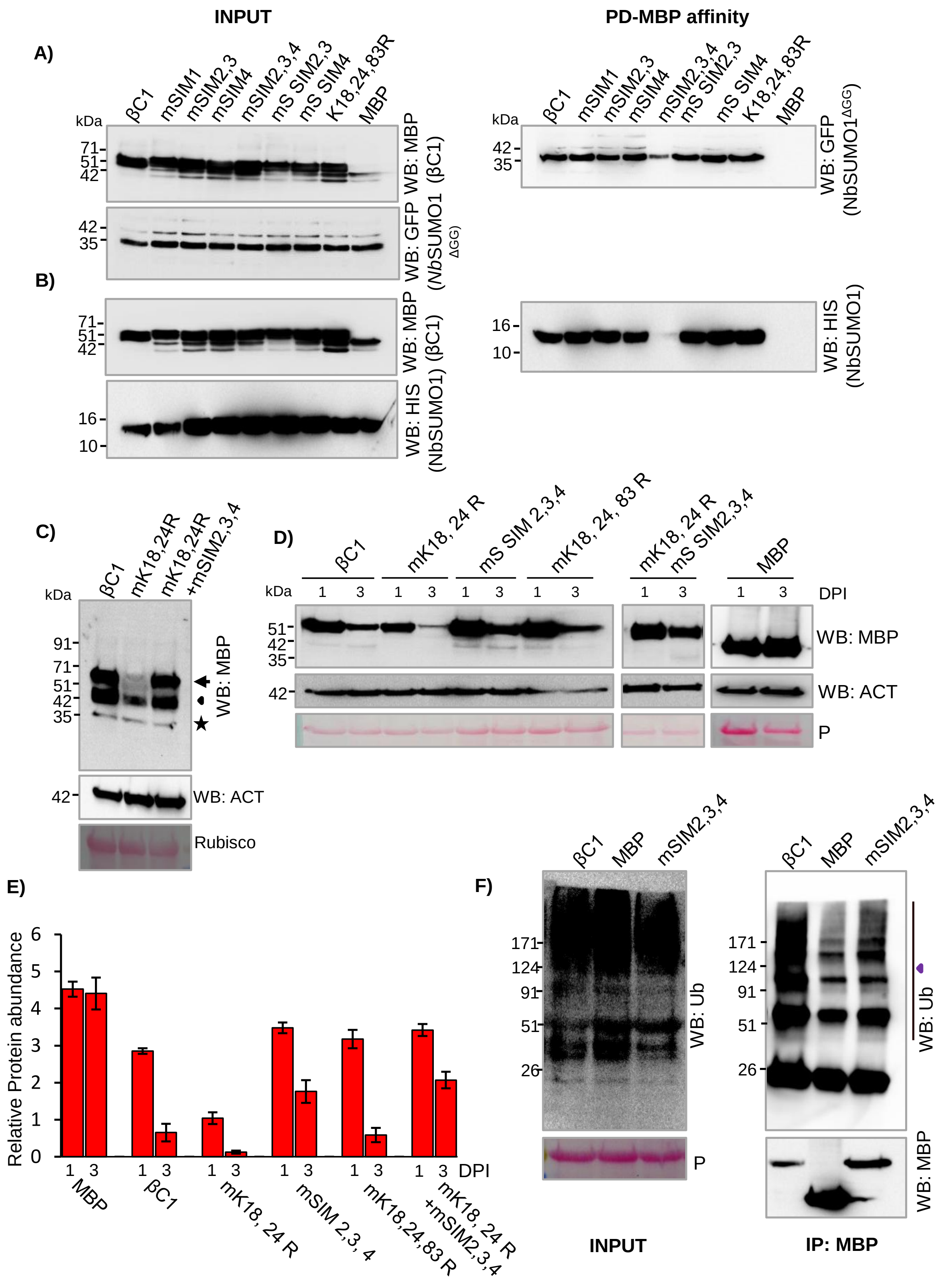

Fig.S12: SIM sites in SyYVCV βC1 regulate its stability.

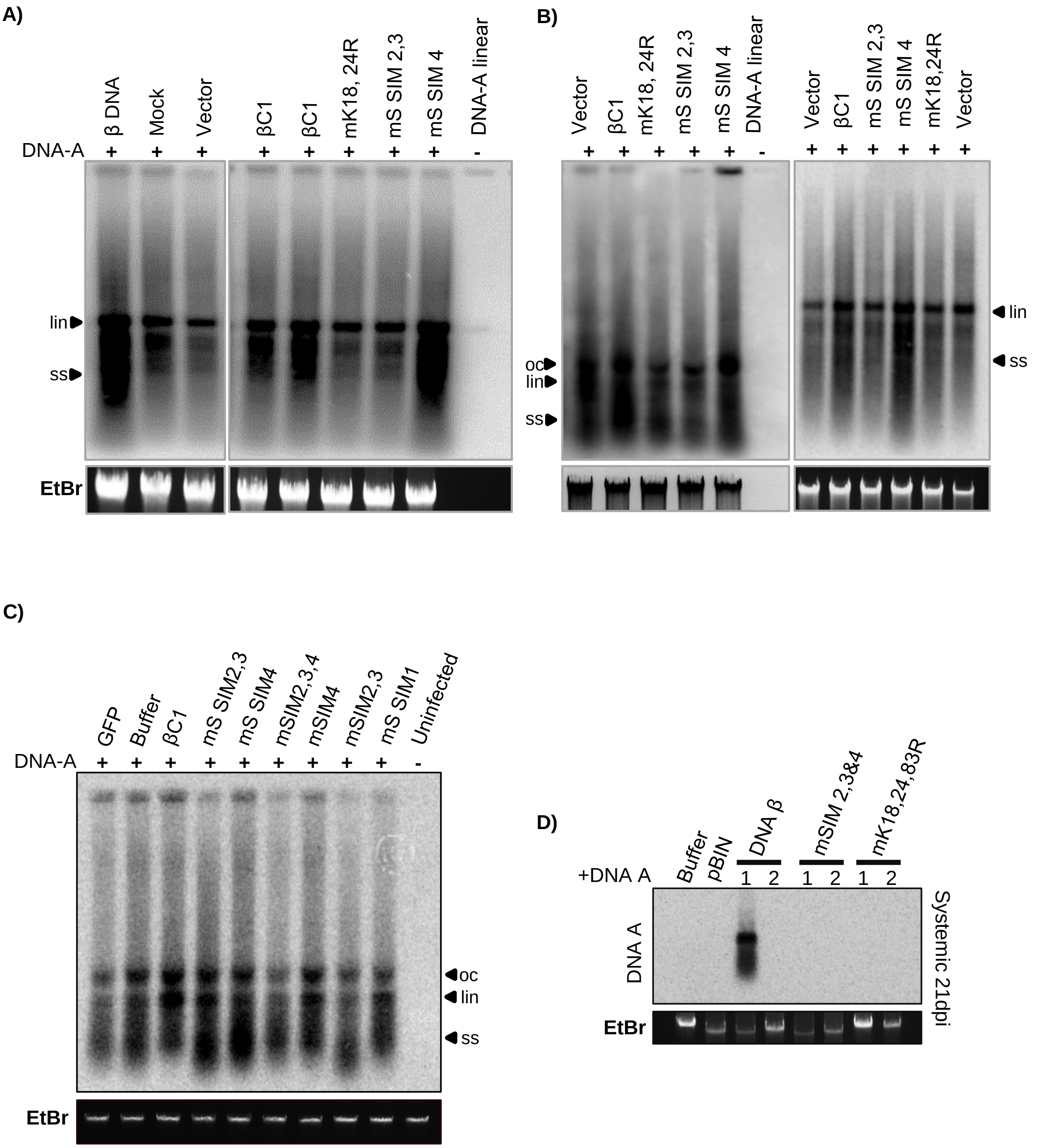

Fig.S13: SIM and SUMOylation motifs of βC1 are necessary for its pathogenicity determinant function.

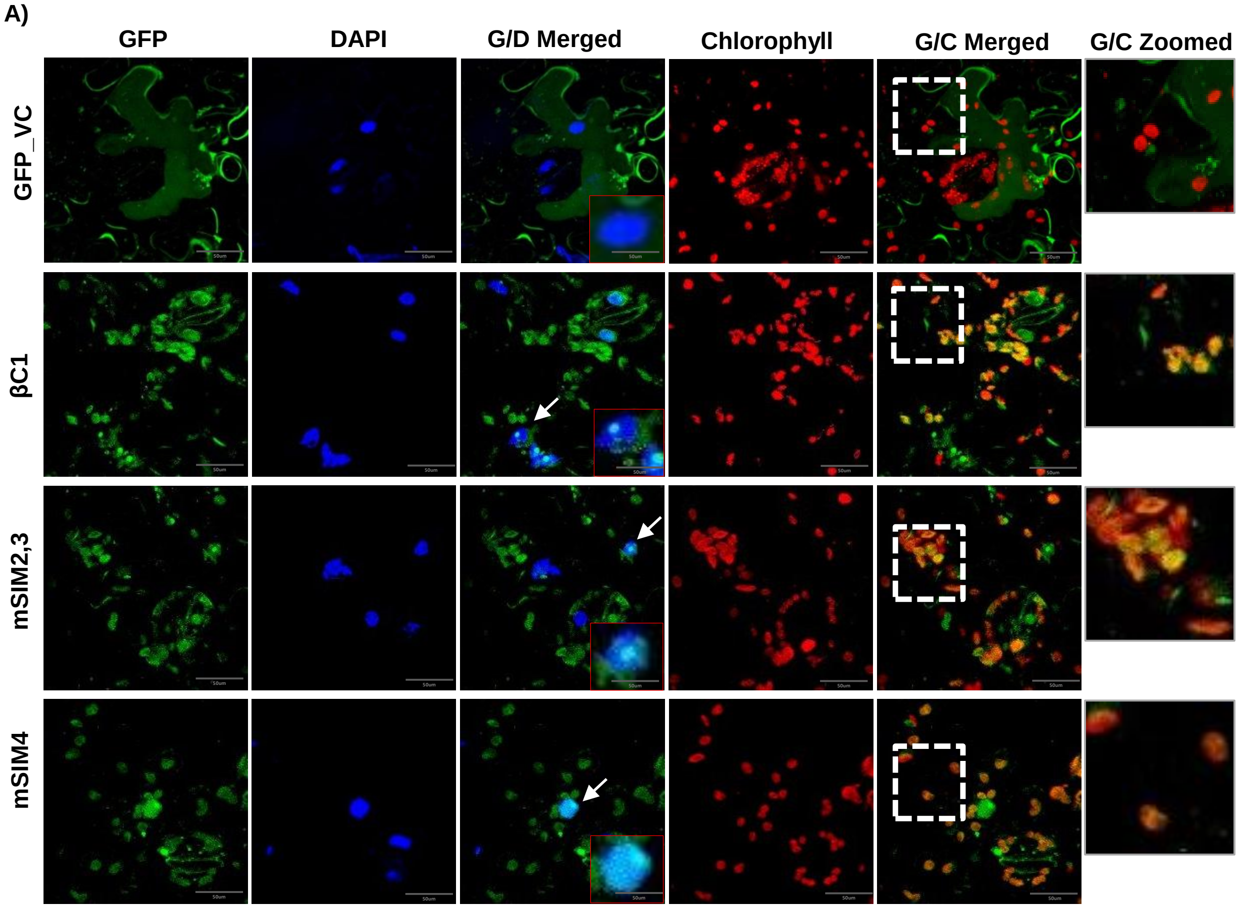

Fig.S14: SUMOylation motif in βC1 is important for its subcellular localization.

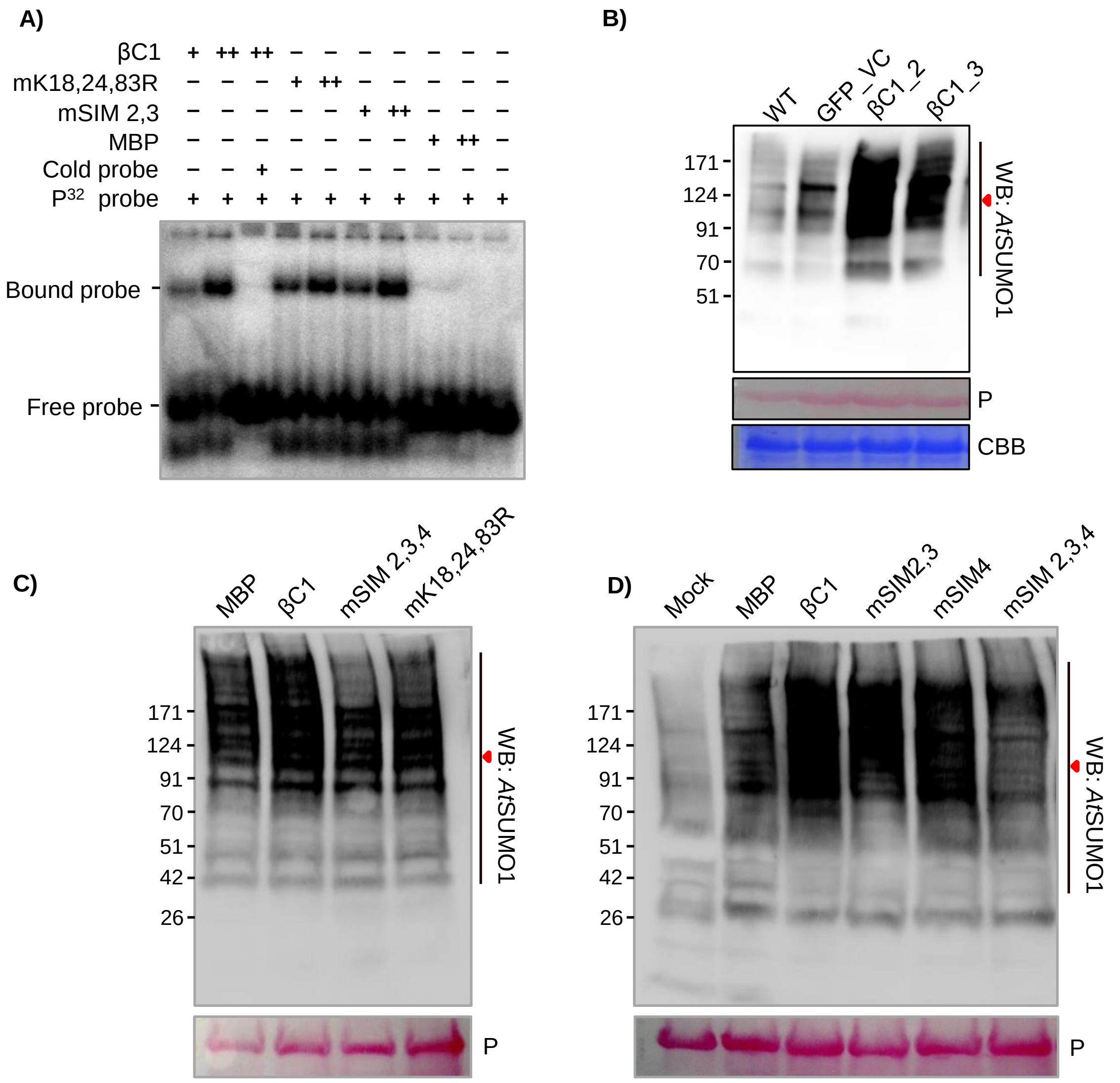

Fig.S15: SyYVCV βC1 induces global SUMOylation.

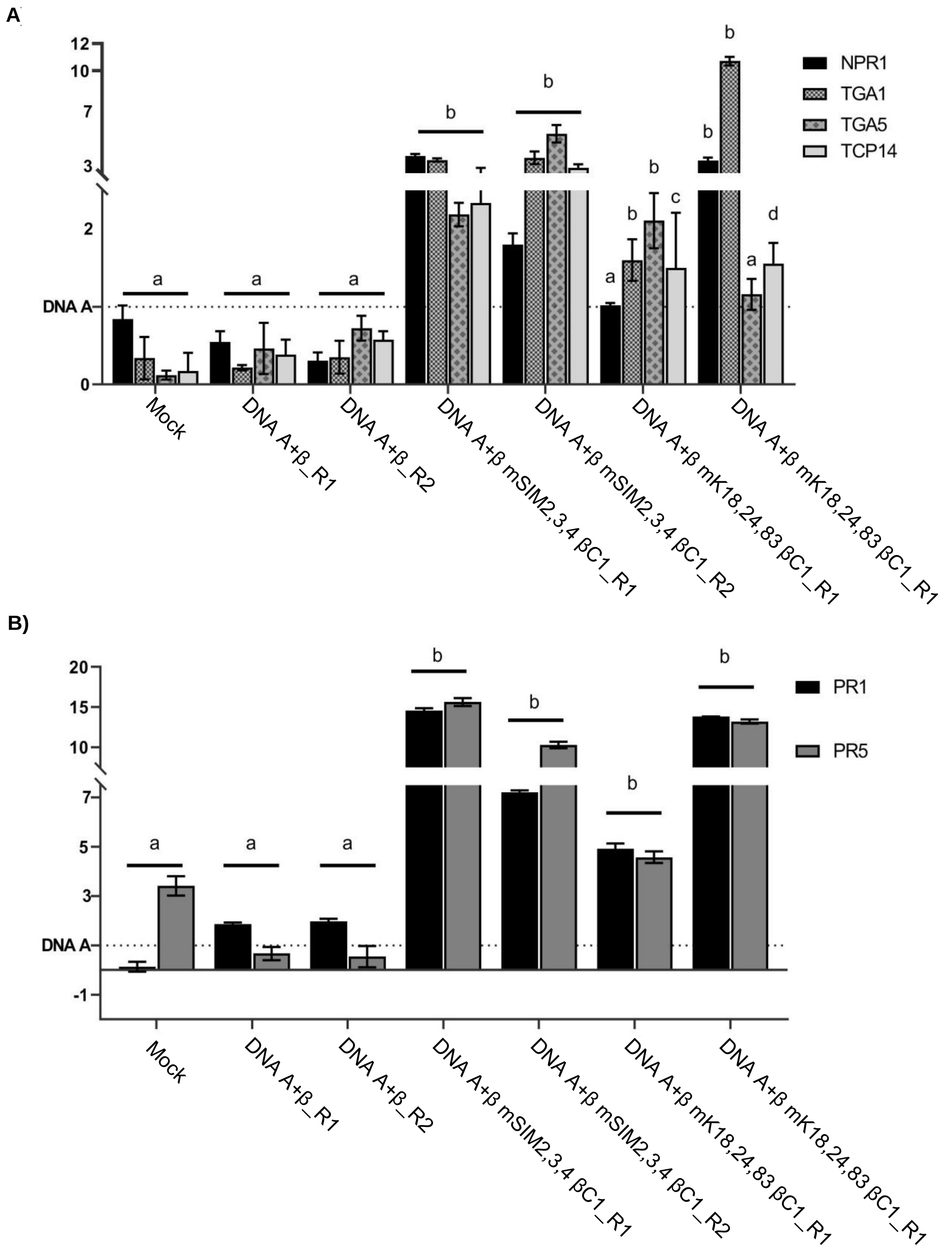

Fig.S16: SIM and SUMOylation motifs of βC1 is essential for host defense suppression.

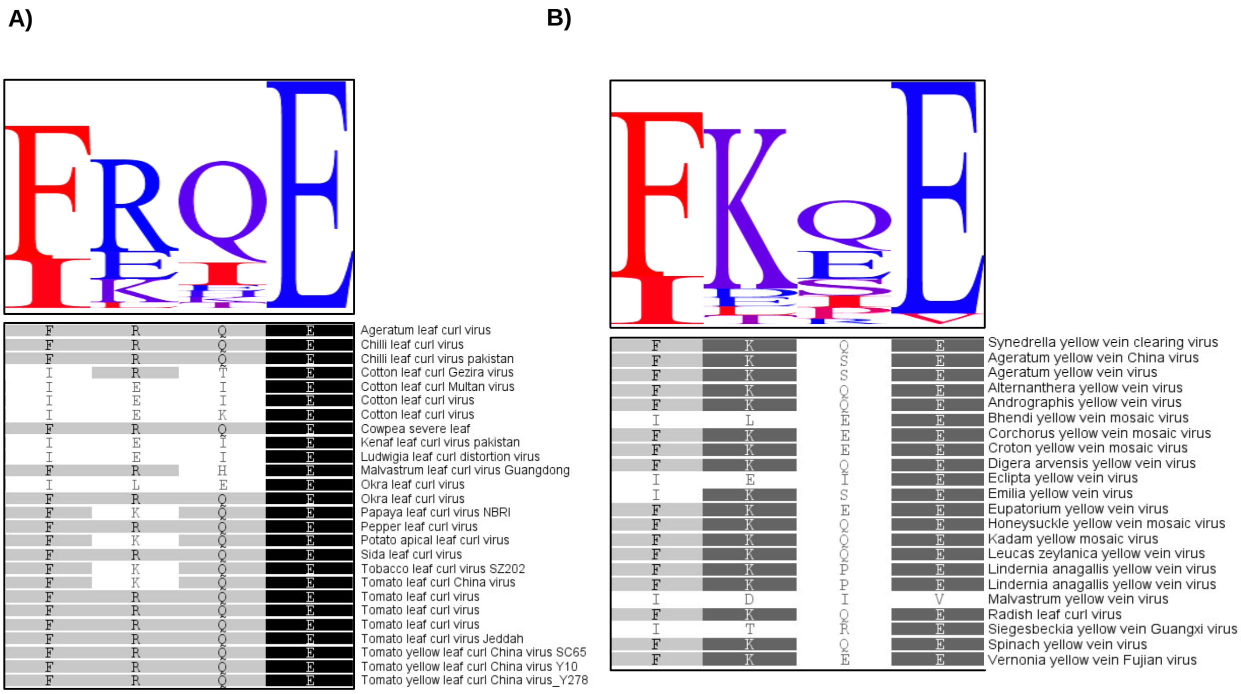

Fig.S17: Conservation of SUMOylation sites among viruses producing similar symptoms..

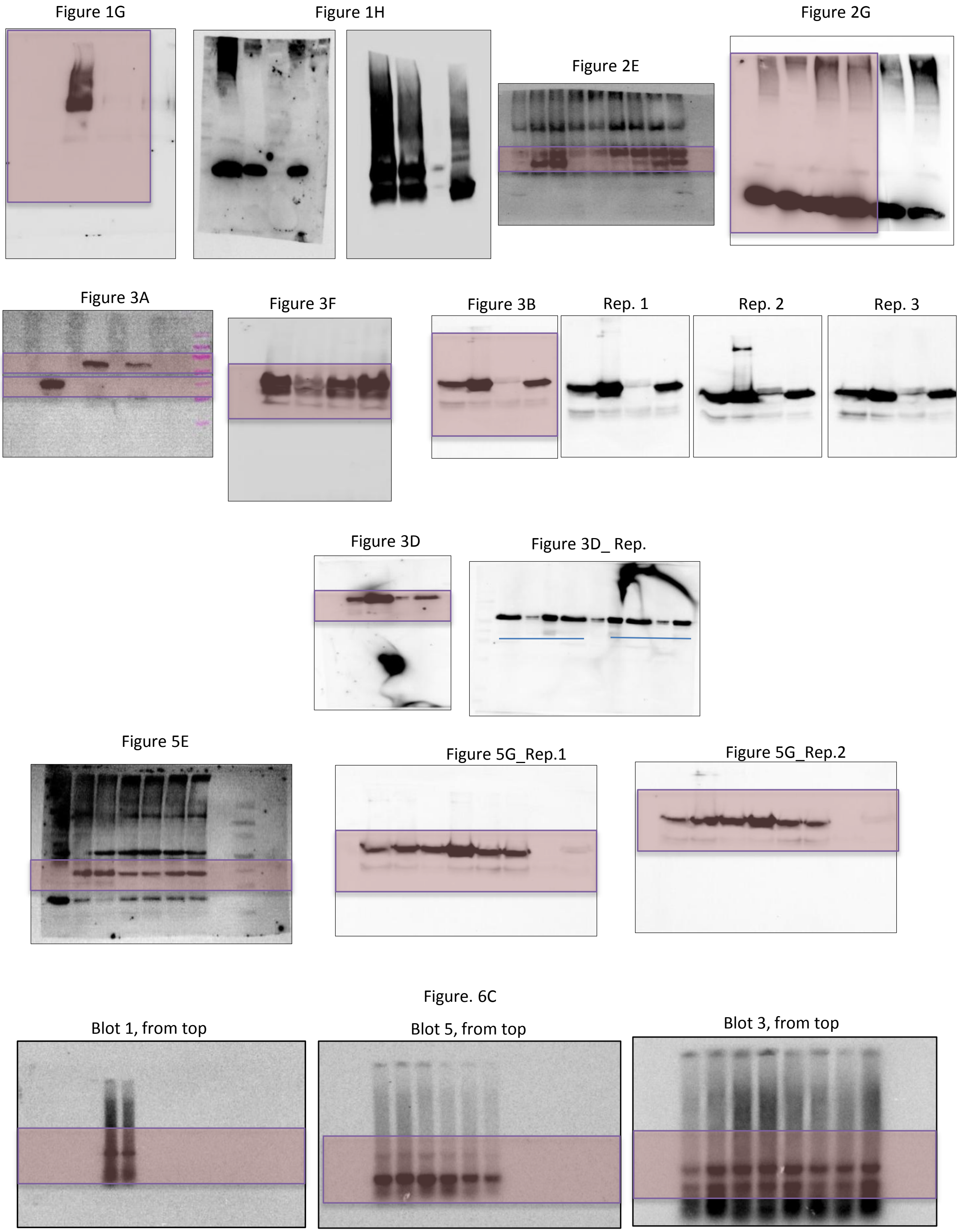

Fig.S18: Uncropped images of blots in main figures 1-7 and replicates of western blots.

S. Figure 4A

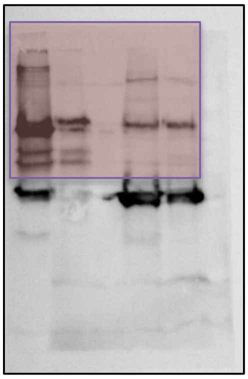

S. Figure 4B

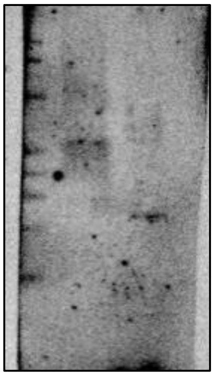

S. Figure 7A

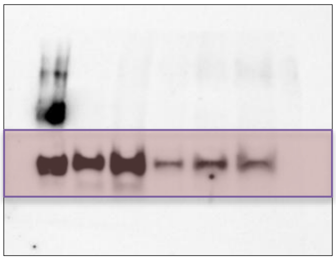

S. Figure 7B

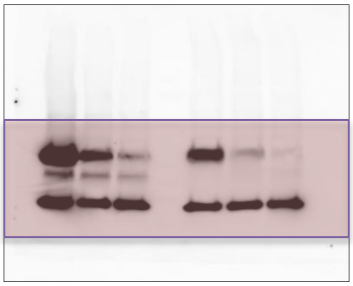

S. Figure 7D

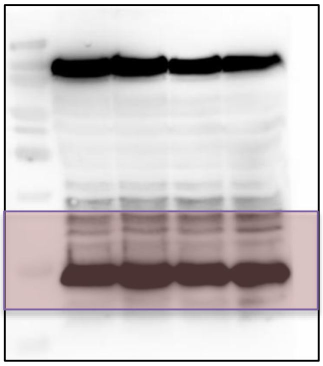

S. Figure 12A

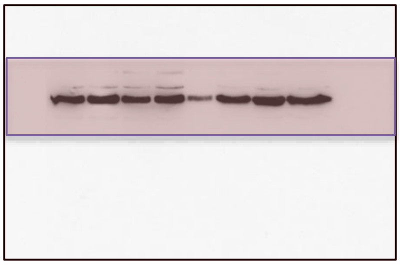

S. Figure 12B

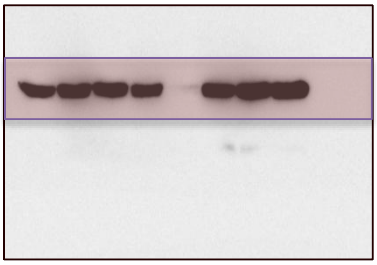

S Figure 12D

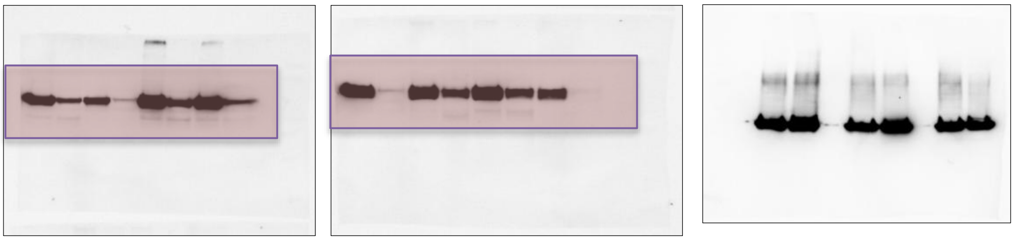

Fig.S19: Uncropped images of blots in Supplemental Figure 1-16.
