# Supplementary material for: Stability of Begomoviral pathogenicity determinant βC1 is modulated by mutually antagonistic SUMOylation and SIM interactions
Source: BMC Biol. 2020 Aug 31;18:110. doi: 10.1186/s12915-020-00843-y (PMC7461331; doi:10.1186/s12915-020-00843-y)
Supplement: Supplementary file 2 — Additional file 2: Table S1: Accession numbers used for building alignments. Table S2: Accession numbers used for K83 site analysis. Table S3: List of primers used in this study. Table S4: List of antibodies and materials used for IP. [file 12915_2020_843_MOESM2_ESM.docx]

**Additional File 2:**

**Table S1: Accession numbers used for building alignments.**

| Accession No. | Accession No. | Accession No. | Accession No. |
| --- | --- | --- | --- |
| TLCVtr\|C6F1P4\|C6F1P4_9VIRU | tr\|D2X8W6\|D2X8W6_9VIRU | tr\|M5BEZ3\|M5BEZ3_9VIRU | tr\|Q80BH1\|Q80BH1_9VIRU |
| tr\|A1E297\|A1E297_9VIRU | tr\|D2X8W8\|D2X8W8_9VIRU | tr\|M5BEZ4\|M5BEZ4_9VIRU | tr\|Q805R8\|Q805R8_9VIRU |
| tr\|A5A4S0\|A5A4S0_9VIRU | tr\|D2X8X0\|D2X8X0_9VIRU | tr\|M5BF86\|M5BF86_9VIRU | tr\|Q807X1\|Q807X1_9VIRU |
| tr\|A5H1F3\|A5H1F3_9VIRU | tr\|D2X8X3\|D2X8X3_9VIRU | tr\|M5BF87\|M5BF87_9VIRU | tr\|Q807X2\|Q807X2_9VIRU |
| tr\|A5H1F7\|A5H1F7_9VIRU | tr\|D3K4G2\|D3K4G2_9VIRU | tr\|Q1JRK2\|Q1JRK2_9VIRU | tr\|Q807X4\|Q807X4_9VIRU |
| tr\|A5H1F8\|A5H1F8_9VIRU | tr\|D5GQ88\|D5GQ88_9VIRU | tr\|Q1JRK4\|Q1JRK4_9VIRU | tr\|Q807X6\|Q807X6_9VIRU |
| tr\|A5H1G0\|A5H1G0_9VIRU | tr\|E2CT18\|E2CT18_9VIRU | tr\|Q1JRK8\|Q1JRK8_9VIRU | tr\|Q807X7\|Q807X7_9VIRU |
| tr\|A6MHR0\|A6MHR0_9VIRU | tr\|E2FHH3\|E2FHH3_9GEMI | tr\|Q1JRK9\|Q1JRK9_9VIRU | tr\|Q807X8\|Q807X8_9VIRU |
| tr\|A8CFR4\|A8CFR4_9VIRU | tr\|E2FHH4\|E2FHH4_9GEMI | tr\|Q1JRL2\|Q1JRL2_9VIRU | tr\|R4NHT7\|R4NHT7_9GEMI |
| tr\|A8W3N3\|A8W3N3_9VIRU | tr\|E3U9R0\|E3U9R0_9VIRU | tr\|Q1JRL3\|Q1JRL3_9VIRU | tr\|R4U4C0\|R4U4C0_9VIRU |
| tr\|A8W3N4\|A8W3N4_9VIRU | tr\|E3U9R1\|E3U9R1_9VIRU | tr\|Q1JRL9\|Q1JRL9_9VIRU | tr\|R9RZM6\|R9RZM6_9VIRU |
| tr\|A8W3N5\|A8W3N5_9VIRU | tr\|E3U9R3\|E3U9R3_9VIRU | tr\|Q1JRM0\|Q1JRM0_9VIRU | tr\|R9RZY6\|R9RZY6_9VIRU |
| tr\|A9NJ54\|A9NJ54_9VIRU | tr\|E3U9R6\|E3U9R6_9VIRU | tr\|Q1JRM1\|Q1JRM1_9VIRU | tr\|S4TLI9\|S4TLI9_9VIRU |
| tr\|A9XCM1\|A9XCM1_9VIRU | tr\|E3U9R8\|E3U9R8_9VIRU | tr\|Q2L9X5\|Q2L9X5_9VIRU | tr\|S5QET7\|S5QET7_9VIRU |
| tr\|A9YSI7\|A9YSI7_9VIRU | tr\|E3U9R9\|E3U9R9_9VIRU | tr\|Q2MCW1\|Q2MCW1_9VIRU | tr\|S5RWE2\|S5RWE2_9VIRU |
| tr\|B0FYN8\|B0FYN8_9VIRU | tr\|E3U9S0\|E3U9S0_9VIRU | tr\|Q2XSV7\|Q2XSV7_9VIRU | tr\|T1QDI8\|T1QDI8_9VIRU |
| tr\|B1A906\|B1A906_9VIRU | tr\|E3VQ47\|E3VQ47_9VIRU | tr\|Q3L1B1\|Q3L1B1_9VIRU | tr\|T1QDR2\|T1QDR2_9VIRU |
| tr\|B2KSE6\|B2KSE6_9VIRU | tr\|E3VQ48\|E3VQ48_9VIRU | tr\|Q4GWU6\|Q4GWU6_9VIRU | tr\|V5K416\|V5K416_9VIRU |
| tr\|B2KSE7\|B2KSE7_9VIRU | tr\|E3VQ50\|E3VQ50_9VIRU | tr\|Q4GWU7\|Q4GWU7_9VIRU | tr\|W5RMQ3\|W5RMQ3_9VIRU |
| tr\|B2LUM1\|B2LUM1_9VIRU | tr\|E5LCF8\|E5LCF8_9VIRU | tr\|Q4GXX2\|Q4GXX2_9VIRU | tr\|W5RNV5\|W5RNV5_9VIRU |
| tr\|B7FBH4\|B7FBH4_9VIRU | tr\|E7CZY3\|E7CZY3_9VIRU | tr\|Q4GXX3\|Q4GXX3_9VIRU | tr\|W8FUW7\|W8FUW7_9VIRU |
| tr\|B7ZDK2\|B7ZDK2_9VIRU | tr\|G0T4T8\|G0T4T8_9VIRU | tr\|Q4GXX4\|Q4GXX4_9VIRU | tr\|X2L628\|X2L628_9VIRU |
| tr\|B7ZDP2\|B7ZDP2_9VIRU | tr\|G1C2P9\|G1C2P9_9VIRU | tr\|Q4GZI3\|Q4GZI3_9VIRU | tr\|X2LD19\|X2LD19_9VIRU |
|  |  |  |  |
| tr\|B7ZDP3\|B7ZDP3_9VIRU | tr\|G3FEW3\|G3FEW3_9VIRU | tr\|Q4QZ56\|Q4QZ56_9VIRU | tr\|A0A0A1I7B8\|A0A+A3:A440A1I7B8_9VIRU |
| tr\|B7ZDS6\|B7ZDS6_9VIRU | tr\|G4V2V0\|G4V2V0_9VIRU | tr\|Q4QZ57\|Q4QZ57_9VIRU | tr\|A0A0A7H9B5\|A0A0A7H9B5_9VIRU |
| tr\|B7ZDS7\|B7ZDS7_9VIRU | tr\|H6UF74\|H6UF74_9VIRU | tr\|Q4VZ10\|Q4VZ10_9VIRU | tr\|A0A0A7HCF2\|A0A0A7HCF2_9VIRU |
| tr\|B7ZDT0\|B7ZDT0_9VIRU | tr\|H6UMH8\|H6UMH8_9VIRU | tr\|Q4VZ12\|Q4VZ12_9VIRU | tr\|A0A0A8TS24\|A0A0A8TS24_9VIRU |
| tr\|B7ZDT2\|B7ZDT2_9VIRU | tr\|H9LCJ7\|H9LCJ7_9VIRU | tr\|Q4VZ13\|Q4VZ13_9VIRU | tr\|A0A0D3LS44\|A0A0D3LS44_9VIRU |
| tr\|B7ZDT3\|B7ZDT3_9VIRU | tr\|H9LCS7\|H9LCS7_9VIRU | tr\|Q4VZ15\|Q4VZ15_9VIRU | tr\|A0A0D3LSN0\|A0A0D3LSN0_9VIRU |
| tr\|C0IY45\|C0IY45_9VIRU | tr\|H9LDH6\|H9LDH6_9VIRU | tr\|Q5DKN5\|Q5DKN5_9VIRU | tr\|A0A0D5K7Y5\|A0A0D5K7Y5_9VIRU |
| tr\|C0M635\|C0M635_9VIRU | tr\|I1VE63\|I1VE63_9VIRU | tr\|Q6EV88\|Q6EV88_9VIRU | tr\|A0A0G2SKG1\|A0A0G2SKG1_9VIRU |
| tr\|C1IHT8\|C1IHT8_9VIRU | tr\|I1VE64\|I1VE64_9VIRU | tr\|Q6T3A0\|Q6T3A0_9VIRU | tr\|A0A0G3Z969\|A0A0G3Z969_9VIRU |
| tr\|C1L384\|C1L384_9VIRU | tr\|I2BJB0\|I2BJB0_9VIRU | tr\|Q6T3A1\|Q6T3A1_9VIRU | tr\|A0A0H4CQT4\|A0A0H4CQT4_9VIRU |
| tr\|C3VIZ3\|C3VIZ3_9VIRU | tr\|I3QKQ4\|I3QKQ4_9VIRU | tr\|Q6ZX26\|Q6ZX26_9VIRU | tr\|A0A0M4MH51\|A0A0M4MH51_9VIRU |
| tr\|C9DRK0\|C9DRK0_9VIRU | tr\|J9S2U1\|J9S2U1_9VIRU | tr\|Q6ZX28\|Q6ZX28_9VIRU | tr\|A0A0N7CX76\|A0A0N7CX76_9VIRU |
| tr\|D0R0G7\|D0R0G7_9VIRU | tr\|K7WN78\|K7WN78_9VIRU | tr\|Q6ZX91\|Q6ZX91_9VIRU | tr\|A0A0N9E6U1\|A0A0N9E6U1_9VIRU |
| tr\|D1YST4\|D1YST4_9GEMI | tr\|L0EJ23\|L0EJ23_9VIRU | tr\|Q7T3Y8\|Q7T3Y8_9VIRU | tr\|A0A0N9E717\|A0A0N9E717_9VIRU |
| tr\|D2DEL1\|D2DEL1_9VIRU | tr\|L0EKB5\|L0EKB5_9VIRU | tr\|Q7T3Y9\|Q7T3Y9_9VIRU | tr\|A0A0P0CBX4\|A0A0P0CBX4_9VIRU |
| tr\|D2KBZ4\|D2KBZ4_9VIRU | tr\|L0EKB9\|L0EKB9_9VIRU | tr\|Q7T3Z7\|Q7T3Z7_9VIRU | tr\|A0A0P1HPM6\|A0A0P1HPM6_9VIRU |
| tr\|D2N1S1\|D2N1S1_9VIRU | tr\|L0EKK7\|L0EKK7_9VIRU | tr\|Q7T6Q5\|Q7T6Q5_9VIRU | tr\|A0A0S2GJU7\|A0A0S2GJU7_9VIRU |
| tr\|D2N1T4\|D2N1T4_9GEMI | tr\|L0EKL2\|L0EKL2_9VIRU | tr\|Q7T474\|Q7T474_9VIRU | tr\|A0A0S2IAD6\|A0A0S2IAD6_9VIRU |
| tr\|D2N2Q3\|D2N2Q3_9VIRU | tr\|L0EL12\|L0EL12_9VIRU | tr\|Q7T476\|Q7T476_9VIRU | tr\|A0A0S2RSF9\|A0A0S2RSF9_9VIRU |
| tr\|D2N147\|D2N147_9VIRU | tr\|L0EL17\|L0EL17_9VIRU | tr\|Q7T477\|Q7T477_9VIRU | tr\|A0A0S2ZXU3\|A0A0S2ZXU3_9VIRU |
| tr\|D2N148\|D2N148_9VIRU | tr\|L0ELQ5\|L0ELQ5_9VIRU | tr\|Q7T480\|Q7T480_9VIRU | tr\|A0A0S3WRI5\|A0A0S3WRI5_9VIRU |
| tr\|D2N284\|D2N284_9VIRU | tr\|L0ELQ8\|L0ELQ8_9VIRU | tr\|Q7T481\|Q7T481_9VIRU | tr\|A0A0S3WYH2\|A0A0S3WYH2_9VIRU |
| tr\|D2T145\|D2T145_9VIRU | tr\|M1KJU3\|M1KJU3_9VIRU | tr\|Q7T482\|Q7T482_9VIRU | tr\|A0A0U2ZSN0\|A0A0U2ZSN0_9VIRU |
| tr\|D2X8S2\|D2X8S2_9VIRU | tr\|M1XFU4\|M1XFU4_9VIRU | tr\|Q7TEX3\|Q7TEX3_9VIRU | tr\|A0A0U5FSY6\|A0A0U5FSY6_9VIRU |
| tr\|D2X8S3\|D2X8S3_9VIRU | tr\|M1XJC3\|M1XJC3_9VIRU | tr\|Q7TEX4\|Q7TEX4_9VIRU | tr\|A0A0X7ZWN5\|A0A0X7ZWN5_9VIRU |
| tr\|D2X8S4\|D2X8S4_9VIRU | tr\|M1XJD5\|M1XJD5_9VIRU | tr\|Q7TEX5\|Q7TEX5_9VIRU | tr\|A0A024E2Z3\|A0A024E2Z3_9VIRU |
| tr\|D2X8S5\|D2X8S5_9VIRU | tr\|M1XJF9\|M1XJF9_9VIRU | tr\|Q7TEX6\|Q7TEX6_9VIRU | tr\|A0A060A3X1\|A0A060A3X1_9VIRU |
| tr\|D2X8S6\|D2X8S6_9VIRU | tr\|M1XLU7\|M1XLU7_9VIRU | tr\|Q8B315\|Q8B315_9VIRU | tr\|A0A060A8J6\|A0A060A8J6_9VIRU |
| tr\|D2X8S8\|D2X8S8_9VIRU | tr\|M4QSY1\|M4QSY1_9VIRU | tr\|Q8B317\|Q8B317_9VIRU | tr\|A0A060KTJ9\|A0A060KTJ9_9VIRU |
| tr\|D2X8T1\|D2X8T1_9VIRU | tr\|M4UQM4\|M4UQM4_9VIRU | tr\|Q8B319\|Q8B319_9VIRU | tr\|A0A060L705\|A0A060L705_9VIRU |
| tr\|D2X8T2\|D2X8T2_9VIRU | tr\|M5BE66\|M5BE66_9VIRU | tr\|Q8B320\|Q8B320_9VIRU | tr\|A0A075QXQ9\|A0A075QXQ9_9VIRU |
| tr\|D2X8T5\|D2X8T5_9VIRU | tr\|M5BE67\|M5BE67_9VIRU | tr\|Q08EU7\|Q08EU7_9VIRU | tr\|A0A077D2F6\|A0A077D2F6_9VIRU |
| tr\|D2X8T6\|D2X8T6_9VIRU | tr\|M5BE68\|M5BE68_9VIRU | tr\|Q9IEZ7\|Q9IEZ7_9VIRU | tr\|A0A088BBX2\|A0A088BBX2_9VIRU |
| tr\|D2X8T7\|D2X8T7_9VIRU | tr\|M5BEA3\|M5BEA3_9VIRU | tr\|Q70UF2\|Q70UF2_9VIRU | tr\|A0A088BC15\|A0A088BC15_9VIRU |
| tr\|D2X8T8\|D2X8T8_9VIRU | tr\|M5BEA5\|M5BEA5_9VIRU | tr\|Q70UF4\|Q70UF4_9VIRU | tr\|A0A088BC62\|A0A088BC62_9VIRU |
| tr\|D2X8V6\|D2X8V6_9VIRU | tr\|M5BEF9\|M5BEF9_9VIRU | tr\|Q70UF5\|Q70UF5_9VIRU | tr\|A0A088BCD9\|A0A088BCD9_9VIRU |
| tr\|D2X8V7\|D2X8V7_9VIRU | tr\|M5BEG2\|M5BEG2_9VIRU | tr\|Q70UF6\|Q70UF6_9VIRU | tr\|A0A088MYP9\|A0A088MYP9_9VIRU |
| tr\|D2X8W5\|D2X8W5_9VIRU | tr\|M5BEG6\|M5BEG6_9VIRU | tr\|Q70UF8\|Q70UF8_9VIRU | tr\|A0A097GUD2\|A0A097GUD2_9VIRU |
| TLCVtr\|C6F1P4\|C6F1P4_9VIRU | tr\|M5BEZ2\|M5BEZ2_9VIRU | tr\|Q70UF9\|Q70UF9_9VIRU | tr\|A0A127Z1Z4\|A0A127Z1Z4_9VIRU |
| tr\|A1E297\|A1E297_9VIRU | tr\|D2X8W6\|D2X8W6_9VIRU | tr\|M5BEZ3\|M5BEZ3_9VIRU | tr\|Q80BH1\|Q80BH1_9VIRU |

**Table S2: Accession numbers used for K83 site analysis.**

| Accession numbers for Leaf curl virus | Accession numbers for Leaf curl virus |
| --- | --- |
| Ageratum leaf curl virus_lcl\|JQ408217.1_prot_AFI41886.1_1 | Ageratum yellow vein China virus_lcl\|AM048836.1_prot_CAJ15641.1_1 |
| Chilli leaf curl virus_lcl\|EU582020.1_prot_ACE80628.1_1 | Ageratum yellow vein virus_lcl\|EF527824.1_prot_ABP96798.1_1 |
| Chilli leaf curl virus pakistan_lcl\|FN179279.1_prot_CAX51845.1_1 | Alternanthera yellow vein virus_lcl\|NC_009562.1_prot_YP_001285942.1_1 |
| Cotton leaf curl Gezira virus_lcl\|FJ868829.1_prot_ACP41025.1_1 | Andrographis yellow vein virus_lcl\|NC_023876.2_prot_YP_009021873.1_1 |
| Cotton leaf curl Multan virus_lcl\|NC_009535.1_prot_YP_001285479.1_1 | Bhendi yellow vein mosaic virus_lcl\|FR823510.2_prot_CBZ47002.1_1 |
| Cotton leaf curl virus_lcl\|HM140826.1_prot_ADJ57305.1_1 | Corchorus yellow vein mosaic virus_lcl\|NC_020475.1_prot_YP_007517309.1_1 |
| Cotton leaf curl virus_lcl\|NC_003200.1_prot_NP_443746.1_1 | Croton yellow vein mosaic virus_lcl\|KM588256.1_prot_AIY67793.1_1 |
| Cowpea severe leaf_lcl\|NC_006952.1_prot_YP_224228.1_1 | Digera arvensis yellow vein virus_lcl\|KY937949.1_prot_AWA81562.1_1 |
| Kenaf leaf curl virus pakistan_lcl\|FR772083.1_prot_CBY88997.1_1 | Eclipta yellow vein virus_lcl\|GQ478345.1_prot_ADM32427.1_1 |
| Ludwigia leaf curl distortion virus_lcl\|JQ408216.2_prot_AFI41885.1_1 | Emilia yellow vein virus_lcl\|JQ247187.1_prot_AFE55702.1_1 |
| Malvastrum leaf curl virus Guangdong_lcl\|KF912951.1_prot_AHN49651.1_1 | Eupatorium yellow vein virus_lcl\|NC_038677.1_prot_YP_009507807.1_1 |
| Okra leaf curl virus_lcl\|FN432358.1_prot_CBA10388.1_1 | Honeysuckle yellow vein mosaic virus _lcl\|GQ495268.1_prot_ACV92103.1_1 |
| Okra leaf curl virus_lcl\|GQ245761.1_prot_ADG29352.1_1 | Kadam yellow mosaic virus_lcl\|LC192884.1_prot_BAX08667.1_1 |
| Papaya leaf curl virus_lcl\|MF683836.1_prot_AWF94053.1_1 | Leucas zeylanica yellow vein virus_lcl\|NC_013424.1_prot_YP_003264272.1_1 |
| Papaya leaf curl virus NBRI_lcl\|JX987089.2_prot_AFV99525.2_1 | Lindernia anagallis yellow vein virus_lcl\|DQ641715.1_prot_ABG26125.1_1 |
| Pepper leaf curl virus_lcl\|NC_010235.1_prot_YP_001648890.1_1 | Lindernia anagallis yellow vein virus_lcl\|NC_009561.1_prot_YP_001285941.1_1 |
| Potato apical leaf curl virus_lcl\|NC_008605.1_prot_YP_899470.1_1 | Malvastrum yellow vein virus_lcl\|NC_004733.1_prot_NP_835276.1_1 |
| Sida leaf curl virus_lcl\|DQ641711.1_prot_ABG26121.1_1 | Radish leaf curl virus_tr\|A9XCM1\|A9XCM1_9VIRU |
| Tobacco leaf curl virus SZ202_lcl\|LT623996.1_prot_SCW2506_1 | Siegesbeckia yellow vein Guangxi virus_lcl\|AM238695.1_prot_CAJ87656.1_1 |
| Tomato leaf curl China virus_lcl\|JF919829.1_prot_AEQ38925.1_1 | Spinach yellow vein virus_lcl\|KF425298.1_prot_AGZ80146.1_1 |
| Tomato leaf curl virus_lcl\|EU847239.1_prot_ACJ54437.1_1 | Synedrella yellow vein clearing virus_lcl\|KX363444.1_prot_APB54218.1_1 |
| Tomato leaf curl virus_lcl\|GU732206.1_prot_ADF42596.1_1 | Vernonia yellow vein Fujian virus_lcl\|NC_015928.1_prot_YP_004778176.1_1 |
| Tomato leaf curl virus_lcl\|MH816961.1_prot_QCB61297.1_1 | Accession numbers for Leaf curl virus |
| Tomato leaf curl virus Jeddah_lcl\|KT312999.1_prot_ALT06561.1_1 | Ageratum yellow vein China virus_lcl\|AM048836.1_prot_CAJ15641.1_1 |
| Tomato yellow leaf curl China virus_Y278_lcl\|AM980511.1_prot_CAQ35107.1_1 | Ageratum yellow vein virus_lcl\|EF527824.1_prot_ABP96798.1_1 |
| Tomato yellow leaf curl China virus SC65_lcl\|GU199589.1_prot_ACZ71251.1_1 | Alternanthera yellow vein virus_lcl\|NC_009562.1_prot_YP_001285942.1_1 |
| Tomato yellow leaf curl China virus Y10_lcl\|AJ421621.1_prot_CAD13480.1_1 | Andrographis yellow vein virus_lcl\|NC_023876.2_prot_YP_009021873.1_1 |

| Gene/Mutation | Sequence (5’to 3’) | Type |
| --- | --- | --- |
| SyYVCV βC1 | F:5’ATATAGTCGACATGACTATCAAGTACAACAACAAGAAAGGC  R: 5’TTAATGAGCTCTCATACAGATACATTACTATACAC | Cloning |
| SyYVCV βC1 | F: 5’AATGGCAGATCTCCATGGGATCCACTATCAAGTACA  R: 5’ATATAGAGCTCCTCGAGGCGGCCGCTCATACAGAT | Cloning |
| mK18, K24R | ATTTCAGTTCAAATACAGCTATTCTCAACACAATCAGCATCCCTAGCAAA | SDM |
| mK83R | GGAGAATTCCGTCAGGAAGACATGATA | SDM |
| mS SUMO3 | GCCACCATCGGAGAAGGCAAGCAGAACGACATGATAGAAATTGT |  |
| mSIM2,3 | CAGGAAGACATGATAGAAGCAGCAGCAGCAGCAATGATGCAAGAGGCTCCAGTG | SDM |
| mSIM4 | ATGATGCAAGAGGCTCCAGCAGCAGCAGCAAATGTATCCGATGAATAC | SDM |
| mS SIM2,3 | ATTCAAGCAGGAAGACATGATAGAAATTAAAGCCATTCTCAACGGC | SDM |
| mS SIM4 | CAAGAGGCTCCAGTGATATCCGGCAATGTATCCGATGAATAC | SDM |
| *Nb*SUMO*1* | F: GATCGGATCCATGTTGCAAACAGAGGAAG  R: CTGGGAGCTCCTCGAGTCAAACAGTTGAGCCTCCAG | Cloning |
| *At*SUMO*1* | F: ATCTGGATCCATGTCTGCAAACCAGGAGGAAG  R:AATTCTCGAGTCAGGCCGTAGCACCACCACC | Cloning |
| *At*SUMO*3* | F: ACGTGGATCCATGTCTAACCCTCAAGATGAC  R:TGCACTCGAGTTAAAGCCCATTATGATCG | Cloning |
| *At*SUMO*5* | F: ATCTAGGATCCATGGTGAGTTCCACAGACAC  R:TCAAGAGCTCCTCGAGTCAGGAGTGTAAGGACCGCC | Cloning |
| MBP | F: CCTCGTAAGACTTCAGCGCTACG  R: GAAACAGCGATGACCATCAACG | qRT |
| SyYVCV βC1 | F: GACGCGCAGACTAATGGTGG  R: GAATTCTCCGATGGTGGCCTC | qRT |
| NPTII | F: CCTGCTTGCCGAATATCATGGTGG  R: TCAGCAATATCACGGGTAGCCAACG | qRT |
| *GAPDH* | F: AACCTTCTTGGCACCACCCT  R: GCAGTGAACGACCCATTTATCTC | qRT |

**Table S3: List of primers used in this study.**

**Table S4: List of antibodies and materials used for IP.**

| Reagent Type | Material | Catalogue No. | RRID |
| --- | --- | --- | --- |
| Antibody | Anti-GFP | Abcam (ab290) | AB_2313768 |
| Antibody | Anti-MBP | Abcam (ab9084) | AB_306992 |
| Antibody | Anti-Flag | Abcam (125243) | AB_11001232 |
| Antibody | Anti-His | CST (2366) | AB_2115719 |
| Antibody | Anti-AtSUMO1 | Agrisera (AS08 308) | AB_2197943 |
| Antibody | Anti-MYC | Abcam (ab9106) | AB_307014 |
| Antibody | Anti-HA | CST (3724) | AB_2798368 |
| IP | GFP-Trap | Chromotek | AB_2827592 |
| IP | Anti-MBP-Magnetic | NEB (E8037S) | - |
| Beads | MBP(Dextrin Sepharose) | GE (28935597) | - |
| Beads | Ni NTA (Agarose) | Qiagen 30210 | - |
| Column | Superdex 75 increase | GE (29148712) | - |
